# Advancing the science on measurement, determinants, and outcomes of pregnancy and abortion coercion in global settings: a scoping review

**DOI:** 10.3389/fgwh.2026.1854104

**Published:** 2026-10-06

**Authors:** Mengjia Liang, Cassie Landers, Sara E. Casey, Goleen Samari, Rachel Snow, Kelli Stidham Hall

**Affiliations:** 1 Heilbrunn Department of Population and Family Health, Mailman School of Public Health, Columbia University, New York, NY, United States; 2 Programme Division, United Nations Population Fund (UNFPA), New York, NY, United States; 3 Department of Population and Public Health Sciences, Keck School of Medicine, University of Southern California, Los Angeles, CA, United States; 4 Independent Researcher, South Thomaston, ME, United States; 5 Departments of Social, Behavioral, and Population Sciences, School of Public Health and Tropical Medicine, Tulane University, New Orleans, LA, United States

**Keywords:** abortion coercion, birth control sabotage, intimate partner violence, reproductive autonomy, reproductive coercion, sexual and reproductive health and rights

## Abstract

Reproductive coercion—behaviors that interfere with women's autonomous reproductive decision-making—is increasingly recognized but remains unevenly defined and measured across settings. We conducted a scoping review and searched MEDLINE, Scopus, CINAHL, Embase, and the Cochrane Library, for English-language studies from 1995 to April 2023. Of 8,515 records identified, 119 studies met inclusion criteria; 56% were quantitative, 55% were published during 2020–2023, and most were conducted in high-income settings (58% United States; 13% Australia). Conceptualizations evolved from a narrow focus on pregnancy coercion and birth control sabotage toward inclusion of abortion coercion, provider-level dynamics, and experiences during motherhood, though consensus is lacking. Measurement was dominated by the Miller reproductive control scale and adaptations; act-based items for abortion coercion were scarce. Reported prevalence spanned ∼1%–58% depending on timeframe, setting, and measure. Multi-level risk factors included unstable or non-marital partnerships, partner concurrency, co-residence with in-laws, and lower household wealth; partner education and employment were protective in several contexts. Reproductive coercion frequently co-occurred with intimate partner, sexual, and domestic violence yet showed independent associations with reduced contraceptive self-efficacy and use, increased covert contraception, unintended pregnancy, sexually transmitted infection, and adverse mental health. Evidence on interventions centers on clinic-based, trauma-informed education and counselling, with implementation barriers related to training, time, and referral pathways. Advancing the field requires a harmonized, intent-sensitive, act-based definition and improved measures, especially for abortion coercion, together with more diverse research in low- and middle-income countries and coordinated legal, health, educational, and community responses to protect women's reproductive autonomy.

## 1 Introduction

Reproductive coercion lies at the intersection of two global commitments affirmed by the 1994 International Conference on Population and Development and reflected in Sustainable Development Goal 5 and its Targets on advancing sexual and reproductive health and reproductive rights and eliminating violence against women and girls (1–3). Within this framework, reproductive coercion is increasingly recognized as a distinct, gendered threat to women's reproductive autonomy, yet it remains comparatively understudied and inconsistently defined and measured across settings.

Reproductive coercion is an understudied and concealed form of violence against women that specifically related to women's sexual and reproductive experiences. Reproductive Coercion arises from the convergence of issues of sexual and reproductive health and rights and violence against women and poses a direct threat to reproductive autonomy and rights. Reproductive coercion refers to behaviors that interfere with autonomous decision-making regarding reproductive health (4). Tarzia and Hegarty suggest that reproductive coercion is the “physical, psychological or sexual violence harnessed for the express intent of promoting or preventing pregnancy” (5). Current literature indicates that reproductive coercion is usually perpetrated by an intimate partner in the domestic United States or a family member in certain international setting (4, 6). It may take in the forms of pregnancy coercion, contraceptive sabotage, and abortion coercion (4). Pregnancy coercion refers to behaviors intended to pressure a woman into becoming pregnant or continuing a pregnancy against her wishes; birth control sabotage refers to direct interference with a woman's contraceptive use, such as hiding, damaging, or removing contraceptives or refusing to use condoms; and abortion coercion refers to attempts to control the outcome of a pregnancy by pressuring or forcing a woman either to terminate or to continue a pregnancy against her wishes (4, 7).

Studies from the United States have suggested that the prevalence of reproductive coercion in the previous three months ranges from 5.1% (8–10) to 13.5% (11); lifetime estimates of reproductive coercion range from 7.8% (12) to a moderate rates of 16% (13); the highest rates of lifetime reproductive coercion were 24% (14) and 29.6% (15). These varied estimates may be due to differences in the populations and study designs, including differences in reference period, recall period, measurement instrument, sampling frame, and the definition of reproductive coercion applied. In international settings, male partner perpetration (18.5%) is generally more common than in-law perpetration (6%) (16, 17). In-law perpetration, documented primarily in South Asian and some sub-Saharan African settings, can involve behaviors distinct from partner perpetration, such as pressure to conceive soon after marriage, son preference, and surveillance or restriction of a woman's contraceptive use (16, 18, 19). Like any other types of violence against women, estimates of reproductive coercion is likely underestimates of women's actual experiences.

Although research on reproductive coercion has grown rapidly since the field emerged in the late 2000s, this growth has been uneven, and mostly concentrated in high-income settings and anchored largely to a single measurement tradition, which leaves persistent gaps in how reproductive coercion is conceptualized, measured, and studied across diverse global contexts. This scoping review aimed to assess the state of the science on reproductive coercion, with a focus on four domains, including conceptualization, multi-level correlates and outcomes associated with reproductive coercion, measurement approaches, and scientific gaps for studying reproductive coercion. The review advanced earlier systematic reviews of this subject (4, 6), synthesizing the latest global evidence, concentrating on a range of constructs related to reproductive coercion, and analyzing multi-level factors that extend beyond the interpersonal level. The review also analyzed the evidence from a life course perspective, with an emphasis on the age differentials and potentially greater vulnerability among adolescent girls and young women. Given the significant gaps in the literature on pregnancy coercion and abortion coercion in LMICs, the review paid particular attention to these forms of reproductive coercion.

## 2 Methods

A scoping review was selected because the aim was to map the breadth and current state of a heterogeneous and still emerging evidence base and to identify conceptual and measurement gaps, rather than to synthesize effect estimates for a narrowly defined question, for which a systematic review would be better suited. The scoping review follows the methodological framework developed by Arksey and O'Malley (20), complemented by the Joanna Briggs Institute (JBI) methodology manual for scoping review (21), and the Preferred Reporting items for Systematic reviews and Meta-analyses (PRISMA-P) guidance for protocol development (22). Specifically, the scoping review included the following five key stages: (1) formulating the research questions; (2) identifying relevant studies; (3) selecting studies; (4) extracting data; (5) collating, summarizing, and reporting the results. This review is reported in accordance with the PRISMA Extension for Scoping Reviews (PRISMA-ScR) (23).

The scoping review was guided by the following research questions, “What is the state of the science, including quality of evidence and strengths, limitations, and gaps in measurement approaches and research designs, on coercion that childbearing-aged women encounter in all aspects of their reproductive experiences and lives, including pregnancy and abortion coercion”; “What are the key dimensions, correlates, and outcomes of reproductive coercion, with attention to pregnancy and abortion coercion, applied in existing measurement approaches and research methodologies used to date?”; and “What are the programme interventions and policy responses have been suggested to date, and what is known about their effectiveness?”.

Electronic searches were conducted for peer-reviewed literature published from January 1, 1995 to April 2, 2023 in English, to capture the full period of formal research on reproductive coercion, the initial search covered 1 January 1995 to 2 April 2023, followed by a supplementary search through September 2023 that identified five additional records. Although the search period began in 1995, the earliest eligible study was published in 2007; thus, the evidence base effectively spans 2007–2023. To reflect the field's rapid growth, we additionally conducted a targeted, non-systematic scan of literature appearing in 2024–2026 and incorporated a small number of highly relevant studies; this scan was not a systematic update of the search, and the systematically searched evidence base is current through 2023. Searches were conducted in MEDLINE through PubMed, Scopus, CINAHL, Embase, and Cochrane Library. Further searches were made on Google Scholar, government and organization websites, and grey literature databases, among others, to locate additional relevant sources. Existing knowledge and networks were utilized, and reference lists from all the included sources were manually searched. The results were exported to Endnote (Clarivate, Philadelphia, PA) for data management and later exported to Covidence (Covidence systematic review software, Veritas Health Innovation, Melbourne, Australia) for removing duplicates, title and abstract screening, full text screening, and data extraction. Studies were included if they meet the inclusion criteria and excluded if they do not meet the criteria as described in Table 1. Briefly, eligible sources were peer-reviewed quantitative and qualitative studies, expert-opinion papers, and grey literature that provided an explicit definition of reproductive coercion (matching or extending Miller et al., 2010), a measurement tool for it, or analyses of its multi-level correlates and outcomes among women in any country and development context. Sources were excluded if they addressed reproductive coercion among men, were single-person case reports, focused on humanitarian settings or specialized groups (e.g., incarcerated women), or were not published in English. Every article was reviewed independently by a pair of reviewers who performed consistency checks to evaluate inter-rater reliability. In case of disagreement, the two reviewers discussed the article's relevancy to determine whether or not it should be included; no formal inter-rater agreement statistic was calculated.

**Table 1:** Inclusion and exclusion criteria.

Domain	Inclusion criteria	Exclusion criteria
Sources	Peer-reviewed studies (quantitative and qualitative) and expert opinion papers, as well as grey literature	Other types of literature.
Focus	Studies and reports on reproductive coercion among women, including conceptualization, measurement approaches, multi-level correlates and outcomes associated with it, and scientific gaps. Studies must provide an explicit definition of reproductive coercion (or its synonyms and/or antonyms) that includes, matches, or goes beyond the definition initially described by Miller et al. (2010), provide a measurement tool for it, or produce analyses of multi-level correlates and outcomes with it.	Reproductive coercion among men and case reports of single individuals was excluded.
Context	General population in all countries and development context	Humanitarian settings, specialized groups such as incarcerated women
Language	English	Other languages
Year	Year limit from 1995	Before 1995
Study design	Observational studies (descriptive and analytical cross-sectional, cohort, case-control), experimental and quasi-experimental studies, and systematic reviews	Other designs (e.g., single-person case reports)

This scoping review includes descriptive observational studies including descriptive cross-sectional studies, and analytical observational studies including prospective and retrospective cohort studies, case-control studies, and analytical cross-sectional studies. Both experimental and quasi-experimental study designs were included. In addition, systematic reviews were considered for inclusion in this scoping review. The initial search terms used select keywords related to reproductive coercion and relevant synonyms and Medical Subject Headings (MeSH) terms (see Supplementary File S1: Search strategy). The database search strings included a filter excluding records indexed with the term “qualitative”, reflecting the original protocol's quantitative focus; the eligibility criteria were later broadened to include qualitative and mixed-methods studies (Table 1) without re-running the searches.

Data extraction was carried out by a single reviewer. Where necessary, authors of the studies were contacted to request any missing details or additional data, as deemed appropriate. The data extraction was structured around a customized table based on the JBI standardized data extraction template.

A comprehensive data extraction table was generated, consolidating all extracted information. Extracted fields included first author and year, country and study setting, study design, participant characteristics, sampling approach and sample size, measurement instrument, the type and socio-ecological level of reproductive coercion, prevalence (overall and by subtype where reported), correlates and risk factors, associations with other forms of violence against women, health outcomes, and interventions or policy responses. The results were synthesized to describe the conceptualization of reproductive coercion, measurement approaches, multi-level correlates and outcomes associated with it, and policy and program implications.

## 3 Results

The search yielded 8,515 potentially relevant citations: 8,421 records identified through the bibliographic databases, and 94 records identified through citation searching and grey-literature sources, of which 89 were identified in the original April 2023 search and 5 were added during May—September 2023. After deduplication and relevance screening, 293 citations met the eligibility criteria based on title and abstract and the corresponding full-text articles were procured for review. After screening of the full-text articles, 119 articles remained relevant and were included in the analysis. The flow of articles through identification to final inclusion is represented in Figure 1.

**Figure 1:**
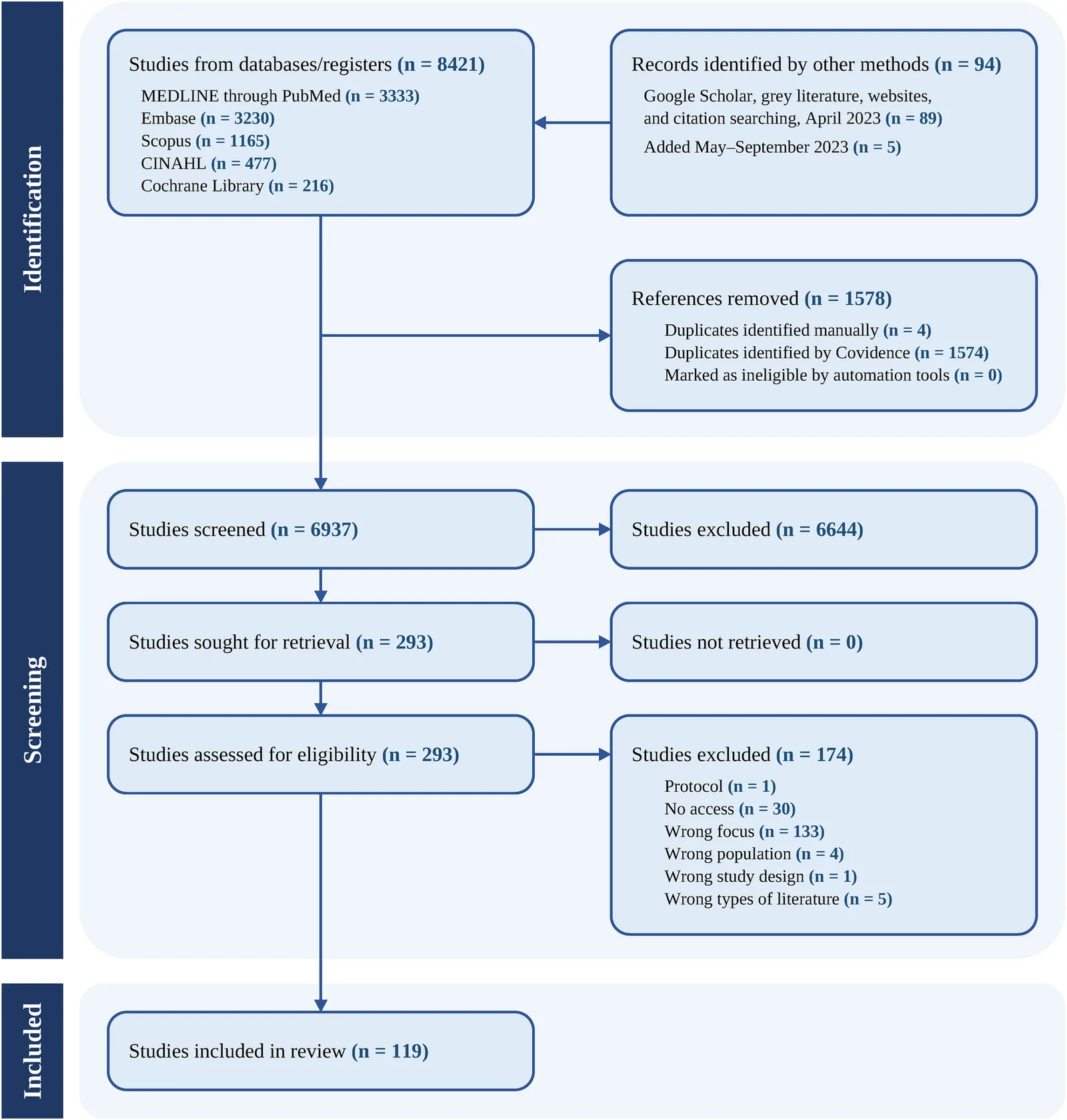
PRISMA-ScR flowchart of study selection process.

The general characteristics of the articles included in this study are reported in Table 2. All included studies were published between 2007 and 2023, with approximately 55% (65/119) published during 2020–2023. More than half of the included studies are quantitative, constituting 56% (66/119) of the total. Geographically, the included studies are predominantly centered on the United States, representing 58% (69/119) of the studies, with Australian studies constituting 13% (15/119). Thematically, there is a notable emphasis on pregnancy coercion and birth control sabotage over abortion coercion or broader reproductive autonomy. Furthermore, the analysis of reproductive coercion is primarily framed at the interpersonal level, mostly involving intimate partners, and to a lesser extent, family members, in contrast to coercion perpetrated by healthcare providers or those manifesting at the community or macrosocial levels.

**Table 2:** General characteristics of included studies (*n* = 119).

Characteristic	Number (*n* = 119)	Percentage (%)
Publication year		
Prior to 2000	0	0.0
2000–2009	2	1.7
2010–2019	52	43.7
2020–2023	65	54.6
Type of study		
Quantitative	66	55.5
Qualitative	31	26.1
Mixed methods	6	5.0
Commentary	6	5.0
Review	9	7.6
Policy document	1	0.8
Country of study		
Australia	15	12.6
Canada	2	1.7
Côte d'Ivoire	2	1.7
England and Wales	1	0.8
Ethiopia	3	2.5
Fiji	1	0.8
India	3	2.5
Jordan	1	0.8
Kenya	2	1.7
Liberia	1	0.8
Mexico	2	1.7
Nepal	1	0.8
Nicaragua	1	0.8
Niger	2	1.7
Nigeria	1	0.8
South Africa	1	0.8
US	69	58.0
Multi-country or no country focus	11	9.2

The presentation of results is structured to align with the research questions, offering an exploration of the current scientific understanding of reproductive coercion as experienced by childbearing-aged women. The following sections address: the conceptualization of reproductive coercion; measurement approaches and their challenges; identified risk factors; associations with women's health outcomes; intersections with other forms of violence against women; women's resistance strategies and interventions designed to combat reproductive coercion. Detailed tables summarizing the data from individual studies are included in Supplementary File S2 to complement the narrative and provide a granular view of the findings. Figure 2 maps how the four review domains relate to the three research questions and the eight thematic findings. Table 3 summarizes the state of the evidence across the eight thematic domains.

**Figure 2:**
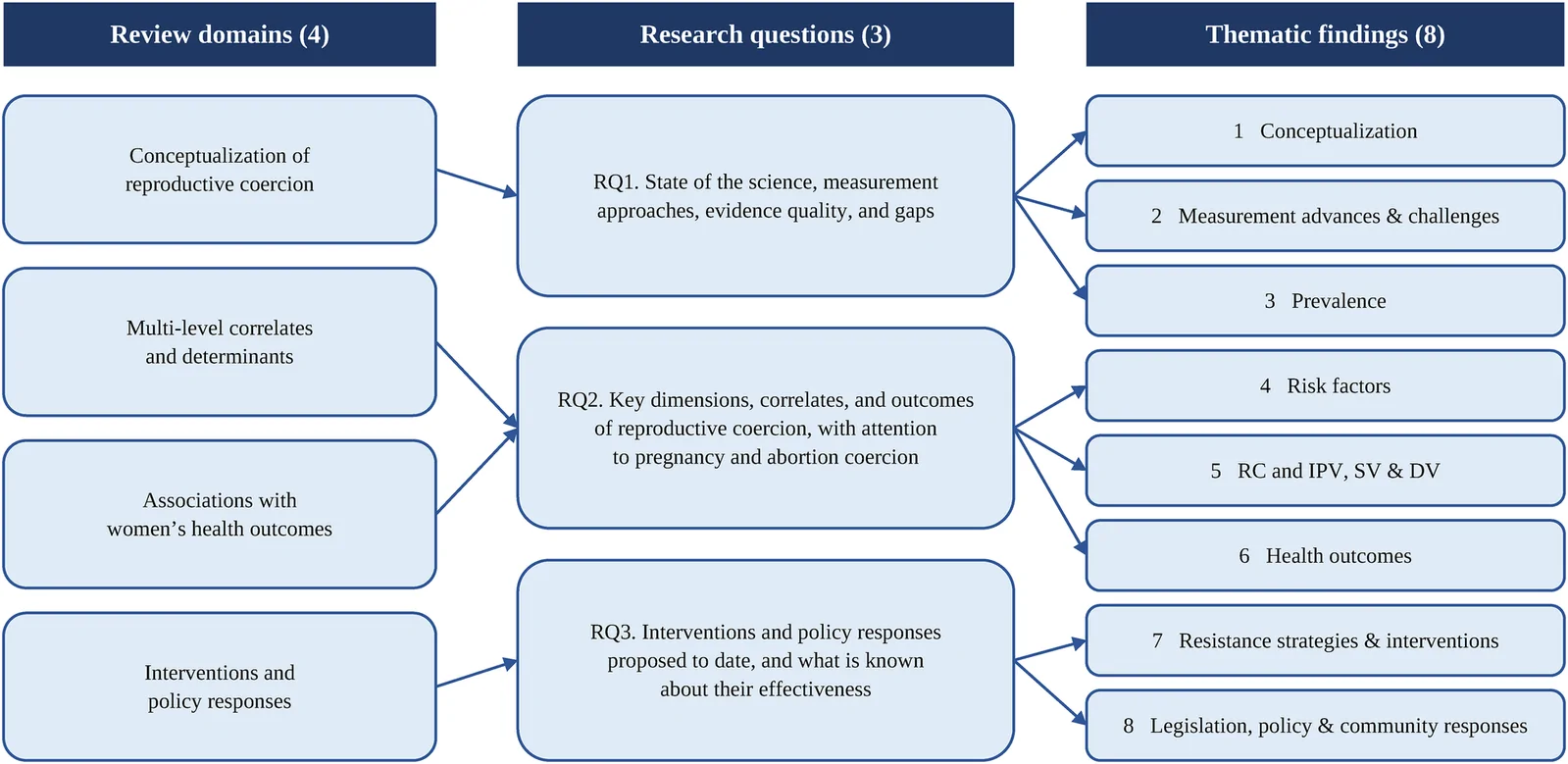
Conceptual framework on review domains, research questions, and thematic findings.

**Table 3:** Summary of the state of the evidence on reproductive coercion, by thematic domain (*n* = 119).

Thematic domain	Studies, *n*^a^	Predominant settings	Key measures/constructs examined	Summary of findings	Key gaps
Conceptualization	9	United States; conceptual and qualitative origins	Definitions of reproductive coercion; subtypes (pregnancy coercion, birth control sabotage, abortion coercion); the intent-vs.-behaviour debate; socio-ecological levels of perpetration	The construct has broadened from pregnancy coercion and birth control sabotage (Miller, 2007/2010) to include abortion coercion and coercion during motherhood; scholars debate whether intent should be definitional (Tarzia) and whether to extend the term to provider and structural levels, with caution about over-inclusiveness.	No consensus on definition or scope; abortion coercion, non-partner perpetration, pregnancy-preventing coercion, and coercion during motherhood remain under-conceptualized.
Measurement	47	United States–dominated; cultural adaptations in LMICs (India, Niger, Côte d'Ivoire, Ethiopia, Burkina Faso, DRC, Kenya, Nigeria, Uganda)	Miller reproductive control scale (pregnancy coercion + birth control sabotage); McCauley 5-item short form; culturally adapted versions adding in-laws and son-preference items; the ARSC scale; provider-based contraceptive-coercion measures (Swan & Cannon, 2024)	The Miller scale and its adaptations dominate and are widely validated and adopted; instruments are diverse but unevenly cover abortion coercion and non-partner perpetrators.	Few validated act-based items for abortion coercion; scarce measures of provider/non-partner coercion; limited cross-cultural validation; no intent dimension in the dominant scale.
Prevalence	41	United States, Australia, and several LMICs (Ethiopia, South Africa, Nicaragua, Kenya); mostly clinic-based samples	Lifetime and past-3-/12-month prevalence; subtype-specific estimates (pregnancy coercion, birth control sabotage, abortion coercion); by sex of respondent	Estimates vary widely (≈1% to ≈58%), reflecting heterogeneity in recall period, instrument, sampling frame, and definition rather than true differences; pregnancy coercion consistently exceeds birth control sabotage, and women report higher rates than men; recent national/population samples extend beyond clinics (Tarzia, 2025; Taylor, 2025).	Few population-based or non-clinic samples; abortion coercion rarely quantified; estimates not comparable across studies; LMICs under-represented.
Risk factors	28	United States, sub-Saharan Africa (Kenya, Ethiopia, Nigeria, Burkina Faso, DRC, Niger), India, Jordan, Nicaragua, Australia	Community, household, couple, and individual-level correlates and determinants	Most consistent: relationship instability, partner concurrency/polygyny, lower household wealth, and in-law cohabitation increase risk, while partner education and employment are protective; mixed and context-dependent for women's age, race/ethnicity, and education; community gender-norm measures inconclusive.	Community and structural determinants under-studied; findings context-dependent and not generalizable; few longitudinal designs to establish temporality.
Reproductive coercion and IPV, SV, and DV	32	United States, Kenya, Australia, Côte d'Ivoire	Co-occurrence and directionality of reproductive coercion with intimate partner, sexual, and domestic violence	Co-occurrence is strong and consistent across settings; reproductive coercion is treated as both a predictor and an outcome of violence, yet it can also occur independently and exerts distinct effects on reproductive health.	Directionality unresolved; evidence largely cross-sectional; the mediating role of reproductive coercion between IPV and health outcomes is under-examined.
Health outcomes	41	United States, Ethiopia, India, South Africa, Australia, Liberia	Contraceptive use and self-efficacy; unintended pregnancy; STI/HIV; maternal and neonatal outcomes; mental and behavioral health	Consistently associated with reduced modern contraceptive use and lower contraceptive self-efficacy, higher unintended pregnancy, higher STI/HIV risk, and adverse mental-health outcomes; effects on specific methods are mixed, partly reflecting covert, female-controlled use.	Causal inference limited by cross-sectional designs; associations with abortion and miscarriage unclear; longitudinal evidence only now emerging.
Women's resistance strategies and interventions	35	United States, Nicaragua, Mexico, Australia, Kenya	Women's coping/resistance strategies; clinical interventions (ARCHES, TIPS); universal education vs. disclosure-based screening	Covert contraception is the most common resistance strategy but rarely secures lasting autonomy; ARCHES and related universal, trauma-informed approaches improve patient and provider confidence, though reductions in reproductive coercion itself are inconsistent.	Few rigorous outcome evaluations; marginalized groups often lack resistance strategies; limited evidence-based guidelines; interventions scarce in LMICs.
Legislation, policy, and community responses	13	Australia (prominent), United States	Legislative recognition within domestic violence frameworks; policy embedding; community and male engagement programmes; technology; support for marginalized groups	Reproductive coercion is often encompassed within legal definitions of domestic violence but is legally complex; authors call for professional training, survivor funding, improved abortion access, norm change and male engagement programmes, and interpreter services for marginalized women.	Little evaluation of policy or legal interventions; perpetrator-focused and community programmes are nascent; limited attention to migrants and structural drivers.

a Studies contribute to more than one thematic domain; the column therefore sums to more than the 119 included studies.

### 3.1 Conceptualization

Behaviors of reproductive coercion were first documented by Miller and colleagues in 2007. The qualitative studies explored reports by adolescent females of male abusive partner pregnancy-promoting behaviors, including condom refusal in setting of perceived pregnancy intention of male partner, condom manipulation associated with pregnancy intention, and birth control sabotage (24). Another qualitative study published in 2010 documented reproductive coercion behaviors that are unrelated to contraceptive methods. The study classified reproductive control along a temporal continuum, including pregnancy promotion and contraceptive sabotage before sexual intercourse; sexual violence, condom manipulation and contraceptive sabotage during sexual intercourse; and controlling pregnancy outcome and interfering with health care post-conception (25). The concept of reproductive coercion was first comprehensively framed by Miller et al. in a 2010 study, which detailed behaviors such as pregnancy coercion by male partners and birth control sabotage—interference with contraceptive measures (7). However, this seminal study did not explicitly use the term “reproductive coercion” nor provide a definition. Subsequently, a systematic review in 2018 was the first among the cited works to offer a definition of reproductive coercion. They described it as “behavior that interferes with the autonomous decision-making of a woman, with regard to reproductive health, taking the form of birth control sabotage, coercion or pressure to get pregnant, or controlling the outcome of a pregnancy” (4). Notably, this review brought abortion coercion into the discussion, advocating for its inclusion in the broader context of reproductive coercion.

In the 2020s, Tarzia and colleagues contributed significantly to the conceptualization of reproductive coercion, advocating for a more precise definition that emphasizes intent and control as fundamental components. The authors argue that the scope of reproductive coercion should not encompass behaviors that, while having reproductive consequences, do not originate from the intent to control reproductive outcomes (5, 26). Examples of such behaviors include “stealthing,” where non-consensual condom removal occurs during intercourse for reasons not related to reproduction, and female genital mutilation, which primarily aims to control women's sexuality rather than their reproductive capabilities (5). Proponents of a refined definition of reproductive coercion assert that incorporating the element of intent within survey measures is essential for enhancing the quality of the evidence base. This precision is believed to yield more accurate prevalence rates, facilitate nuanced analyses of risk factors, and delineate clear associations with reproductive coercion distinct from related issues such as sexual or intimate partner violence. In contrast, some scholars contend that the inclusion of intent is superfluous. They argue for a strictly behavioral approach in measurement, asserting that intention may not be discernible to the women affected, could differ from the perpetrator's actual intent, and may not alter the nature of the interventions required to address the behaviors constitutive of reproductive coercion (15). The majority of existing studies have predominantly focused on pregnancy-promoting behaviors, with insufficient analysis dedicated to the intricacies of whether perpetrators' actions are aimed at promoting or preventing pregnancy. Recent research indicates that a substantial portion of reproductive coercion, including behaviors that discourage or prevent pregnancy, has been overlooked, causing the reported prevalence rates to seem lower than they are in actuality (27).

While there is a growing acknowledgment of reproductive coercion in the forms of pregnancy pressure and contraceptive sabotage, the issue of abortion coercion has often been omitted from the discourse. Pike contends that the research community must acknowledge the existence and relative commonality of abortion coercion. This form of coercion not only results in more adverse outcomes than its absence but also infringes upon women's fundamental right to informed consent (28). Moreover, while research has largely focused on the prenatal period, there is evidence of reproductive coercion extending into birthing and postpartum phases, affecting self-esteem and perceptions of motherhood (29). This phase can be exploited by abusive partners to enforce traditional gender roles and further examination of the links between coercive control during motherhood and reproductive coercion may be beneficial.

The prevailing body of research on reproductive coercion predominantly concentrates on the interpersonal dynamics, often perpetrated by partners or family members, while largely overlooking coercion enacted by healthcare providers or at community and structural levels. However, researchers argued that the term “reproductive coercion” risks becoming overly inclusive when applied to such broad contexts, as it can then encompass any factor that potentially impacts a woman's reproductive choices (5). They argue that structural inequalities and governmental policies, while contributing to an environment conducive to reproductive coercion, should be distinguished from reproductive coercion itself. The notion of a “continuum” of reproductive coercion that spans from intimate relationships to policy-making lacks robust empirical support and requires more rigorous evidence (5).

### 3.2 Measurement advances and challenges

Building on the results of a 2007 qualitative study, Miller and colleagues developed and validated a reproductive control scale in 2010 to explore the associations between reproductive control and partner violence and test risk for unintended pregnancy among young adult females in the United States (7). This is the very first reproductive coercion measure, and one of the most widely cited reproductive coercion scales in the literature. The reproductive control scale (the “Miller Scale”) is designed to represent a boarder array of reproductive control experience among women of reproductive age and is consist of pregnancy coercion and birth control sabotage. Pregnancy coercion is an investigator developed assessment and is defined as a positive answer to any of the 6 items. Birth control sabotage is a 5-item measure based on the authors' previous qualitative work (24). A positive response to any of these 5 items is indicative of birth control sabotage.

Numerous later quantitative studies on reproductive coercion have adopted or adapted the Miller Scale, significantly advancing the study of reproductive coercion. Within the forty-seven studies analyzed in the current review, ten directly implemented the scale, with a primary focus on the United States alongside two LMICs—Nicaragua and Côte d'Ivoire (11–13, 30–37). In a significant development in 2017, McCauley et al. refined the measurement by introducing a shorter version of the reproductive coercion scale comprising just five items. These items are: (1) being told not to use birth control; (2) having birth control removed or access to it obstructed; (3) being coerced into unprotected intercourse with the intent to cause pregnancy; (4) experiencing removal of condoms during intercourse to induce pregnancy; and (5) intentional damage to condoms to induce pregnancy (38). The authors demonstrated that this abbreviated scale is efficient for researchers who need to streamline their instruments while maintaining the ability to identify women across the spectrum of reproductive coercion severity, with solid face validity based on prior research on reproductive coercion. In the current review, nine studies have adopted this shorter measure of reproductive coercion, predominantly in the United States but also extending to countries such as Ethiopia and South Africa (14, 39–46).

The original Miller scale, tailored for adolescents and youth in clinical settings within the United States, has since been adapted by six studies to reflect different cultural contexts, including but not limited to Côte d'Ivoire, India, Niger, Ethiopia, Burkina Faso, the Democratic Republic of Congo (DRC), Kenya, Nigeria, and Uganda (17–19, 47–49). These adaptations often expanded perpetrators to include husbands and in-laws and added context-specific items, such as son-preference pressure or threats of abandonment, informed by local formative work (18, 19). Thirteen studies have substantially modified the Miller scale to meet different research needs (15, 50–61). For example, some studies opted for a broad single-item approach, asking if a woman's partner, including current or former husbands, “tried to keep them from using birth control and getting them pregnant when they did not want to” (53), or “ever pressured you not to use birth control, taken your birth control (like pills away from you, or kept you from going to the clinic to get birth control?” (54). To bridge identified research gaps, other studies incorporated items pertaining to abortion coercion, such as “tried to make you get an abortion; tried to stop you from getting an abortion; violence or threats to try to make you get an abortion; violence or threats of violence to try to stop you from getting an abortion” and distinguished between pregnancy-promoting intentions and coercive actions (58, 62). Not all reproductive coercion measures are related to the Miller scale. Alternative methodologies were developed by other studies to explore reproductive coercion (27, 63–67). One study implemented the Adolescent Reproductive and Sexual Coercion (ARSC) scale, which encompasses five items designed to detect a spectrum of coercive behaviors, both verbal and physical. Additionally, two studies adopted a more open-ended approach, empowering counsellors to identify behaviors indicative of reproductive coercion across its three temporal domains: pressure to achieve pregnancy, interference with contraception, and control over pregnancy outcomes (27, 66).

### 3.3 Prevalence of reproductive coercion

A review of the samples across the examined studies reveals a diverse array of sources: twenty-one utilized clinic-based populations, four drew from community settings, three capitalized on internet-based outreach, six were population-based, eight school-based, four service-based, and one used snowball sampling method. The sample sizes varied substantially, ranging from 70 to 20,252 individuals (42, 53). The majority of the measurements focused on interpersonal dynamics, specifically those involving intimate partners. Only four studies expanded this scope to include reproductive coercion by family members, typically in-laws (17, 18, 47, 63). One study took a broader approach, investigating reproductive coercion at the community level and examining the roles of healthcare providers in addition to partners and family members (64). The timeframes over which reproductive coercion was assessed spanned from the previous 3 months to participants' entire lifetimes. The reported prevalence rates of reproductive coercion were markedly varied, with the lowest being 1.1%, observed in a lifetime study among women aged 17 and above who had recently given birth in the United States, and the highest reaching 57.6% over the past 12 months in a population-based sample of ever-married Ethiopian women aged 18–45 years (53, 59). Narrowing the focus to studies using the Miller scale or its adaptations, the reported rates ranged from 6.3% over the past 3 months in a clinic-based sample of U.S. women aged 16–29 years seeking family planning services, to 30.1% lifetime prevalence in a service-based sample of U.S. women aged 16–24 years connected with youth-serving agencies in the child welfare system, and even 40.8% lifetime prevalence of pregnancy coercion in a clinic-based South African sample of sexually active women aged 15–29 years willing to disclose HIV results (38, 44, 45). When data were separated for pregnancy coercion and birth control sabotage, the former consistently showed higher prevalence rates (7, 12, 13, 31, 37). Furthermore, in studies that included both female and male participants, the rates of reproductive coercion reported by females were significantly higher than those reported by males (60, 65). Although not uniformly reported, the prevalence of abortion coercion was specifically addressed in three studies (57, 58, 66).

### 3.4 Reproductive coercion risk factors

While reproductive coercion varies across different contexts, certain sociodemographic characteristics may predispose women to increased risks (see Table 4). Community and household level risk factors are less studied. A study in Kenya found that a gender norms measure was not significantly associated with reproductive coercion, suggesting that existing measures may not capture the relevant cultural norms (68); at the household level, rural residency, lower household income, and cohabitation with in-laws have each been associated with higher risk (49, 53, 63). At the couple's level, less stable relationships, such as “on again/off again” dynamics, separated, or non-marital cohabitation, are significant risk factors for reproductive coercion (49, 53, 56, 66). Partner contraceptive interference, multiple or concurrent sexual partnerships, transactional sex, and polygyny have likewise been associated with greater risk (14, 40, 43, 49, 54, 68–70). Education and employment status of a partner also correlate with reproductive coercion. Across Nicaragua, Kenya, and Nigeria, women whose partners had more education or were employed had lower odds of coercion (31, 49). A study investigating men's hostile sexism found its associations with attitudes endorsing male authority over female reproductive decisions (71). Evidence on age difference between couples is mixed, while one study suggested that women experiencing reproductive coercion are more than 7 times as likely to have sex with a male partner 5 years or more older, other studies suggest that it is not associated with reproductive coercion (45, 56). While partner alcohol and drug abuse have been examined as potential risk factors for reproductive coercion, the evidence remains inconclusive, with studies reporting mixed associations (56, 57).

**Table 4:** Summary results on reproductive coercion risk factors.

Level	Risk factor	Findings
Household	Place of residence	Rural residency increased likelihood of reporting RC.
Household	Household income	Lower household income and wealth were associated with increased odds of RC, while increased wealth showed a protective effect.
Household	Household composition	Cohabitation with in-laws is associated with increased incidence of pregnancy coercion.
Couple	Relationship status	Being single, in a dating relationship, or uncertain of relationship status, being in an “on again/off again” relationship, having multiple sexual partners, history of transactional sex, or a partner engaged in concurrent relationships or polygyny are risk factors.
Couple	Partner's education and employment	Women whose partners with higher education or employed are protective factors.
Individual	Age	Some studies indicated younger women are more likely to experience RC; others suggested that older age may elevate the risk of RC. A few studies found no association.
Individual	Race/Ethnicity	Many suggested Hispanic or non-Hispanic Black women are at an elevated risk. A few studies found no association.
Individual	Women's education	Higher education correlates with reduced RC risk.
Individual	Women's employment	Employed women reported higher instances of RC.

At the individual level, research presents varied findings regarding the influence of women's age on reproductive coercion. Findings are mixed; several studies found younger women at higher risk (33, 53, 56), with risk increasing with age among adolescents (43), whereas others associated older age with birth control sabotage (66, 70, 72) or found no significant association (14, 56). Several studies have investigated the influence of race and ethnicity on reproductive coercion within various contexts in the United States, Kenya, and Australia. Findings are inconsistent: U.S. studies most often reported elevated risk among Hispanic or non-Hispanic Black women (12, 35, 37, 53, 56, 70, 72), though some found higher rates among White women or lower rates among Latina women (14); an Australian study found higher pregnancy-promoting coercion among Aboriginal and/or Torres Strait Islander women (27), while some U.S. and Kenyan studies found no association (34, 68). Women's reproductive history, including birth, pregnancy, abortion and miscarriage experiences, is likely to be associated to reproductive coercion. Studies suggest that in the United States, Democratic Republic of Congo, Kenya, and Ethiopia, women with more children are generally at a reduced risk of reproductive coercion (42, 48, 49). Women experiencing coercion were also less likely to report a current pregnancy and more likely to report an unwanted prior pregnancy, abortion, or miscarriage (13, 40, 56, 68, 72). Other individual-level factors have also been examined for their associations with reproductive coercion, though the evidence may be less robust. Higher women's education has been associated with lower risk (31, 33, 53), whereas employment status shows mixed associations (14, 31); religious affiliation, pregnancy fatalism, and plurisexual attraction have been linked to risk in specific settings (46, 48, 61, 68, 73).

### 3.5 Reproductive coercion and intimate partner violence, sexual violence, and domestic violence

IPV is one of the most prevalent forms of violence against women on a global scale. The United Nations defines IPV as behavior by a current or former intimate partner that causes physical, sexual, or psychological harm. This includes acts of physical aggression, sexual coercion, psychological abuse, and controlling behaviors. IPV represents one of the manifestations of domestic violence (DV), which encompasses violence within the private sphere, often among individuals linked by blood or intimate relationships. Sexual Violence (SV) refers to any sexual act or attempt at a sexual act performed against a person's sexuality through coercion by anyone, irrespective of their relationship with the victim, across various settings. In this review, IPV, SV, and DV are generally discussed in the context of violence against women perpetrated by partners or family members, unless specified otherwise. The interrelationships among IPV, SV, and DV with reproductive coercion are explored in the literature. While many studies categorize reproductive coercion as a form of violence against women, others suggest that IPV and SV act as mechanisms for reproductive coercion, suggesting that reproductive coercion is rarely found without some other form of co-occurring violence within a relationship, or that reproductive coercion acts as a mediator between IPV and other health outcomes such as HIV (4, 5, 44).

The concurrence of reproductive coercion with IPV, SV, and DV is well-established in various contexts. For example, 38% of young Black women in Baltimore reported both reproductive coercion and IPV (74), over 80% of Kenyan IPV survivors reported recent coercion (68), and 34% of Australian women seeking help for unwanted pregnancy reported concurrent coercion, while only 5.7% reported coercion without other domestic violence (66). The directionality of associations between reproductive coercion and other forms of violence against women, such as IPV, SV, or DV, is not universally agreed upon in the literature and reproductive coercion has been treated as both a predictor and an outcome of violence. However, virtually all studies consistently demonstrate a strong association between reproductive coercion and other forms of violence against women, including IPV, SV, and DV. Women subjected to these types of violence are more likely to also report reproductive coercion (32, 34, 39, 53, 57, 59, 63, 66, 74–76). The disparities in reported instances can be quite stark. Similarly, women reporting reproductive coercion are more likely to report other types of violence against women (15, 30, 33, 34, 37, 42, 45, 50, 51, 60). Research on reproductive coercion highlights its independent impact on women's reproductive health outcomes, distinct from other forms of violence such as IPV, SV, and DV. While studies underscore the strong associations between reproductive coercion and these forms of violence, they also reveal that reproductive coercion can occur independently. Notably, Grace and colleagues found that the majority of women experiencing reproductive coercion did not simultaneously experience IPV, suggesting that reproductive coercion should be considered a related, yet separate phenomenon (57). Further investigations have consistently shown that reproductive coercion contributes to an increased risk of adverse reproductive health outcomes. Controlling for IPV and demographics, reproductive coercion independently elevated the risk of unintended pregnancy and reduced modern contraceptive use, with effects amplified when combined with a history of IPV (10, 18). This body of work calls for recognizing reproductive coercion as a distinct type of violence against women that requires targeted interventions to mitigate its specific impact on women's reproductive rights and health.

### 3.6 Associations between reproductive coercion and women's health outcomes

There is a considerable and growing literature base on associations between reproductive coercion generally and specific reproductive health outcomes. Existing studies suggest strong associations between reproductive coercion and reproductive health outcomes such as modern contraceptive use and unintended pregnancy (see Table 5). Evidence indicates a general trend of reduced modern contraceptive use among women who have experienced reproductive coercion, with adjusted odds ratios ranging from 0.18 to 0.77 across studies from the United States, Ethiopia, and India (15, 18, 43, 47, 48, 59). However, findings on specific contraceptive methods vary. Effects on specific methods varied by setting. Reproductive coercion was associated with lower condom and oral-contraceptive use in the United States and Australia (40, 50), but with greater use of long-acting reversible methods, injectables, IUDs, and emergency contraception in South Africa, India, and the United States; these are patterns consistent with covert, female-controlled use (11, 12, 44, 47). The choice of contraceptive method may be influenced by women's covert use as a strategy against reproductive coercion. Studies suggest that covert contraception is linked to young women's experiences of reproductive coercion (77), although detailed analyses on method, type of coercion, perpetrator, and severity are lacking. In Kenya, a one-unit increase in reproductive coercion score was associated with the increased relative risk of covert contraceptive use (RRR = 1.10) and decreased overt use of contraception compared to nonuse (RRR = 0.89) (78). While not all research establishes a significant association between reproductive coercion and contraceptive or covert contraceptive use, studies consistently demonstrate that reproductive coercion is associated with lower contraceptive self-efficacy (15, 19, 36, 40, 79). This includes diminished intent to purchase or use condoms, increased fear of negotiating condom use, and a perception of greater barriers to condom utilization, leading to reduced confidence in both condom negotiation and overall sexual and contraceptive decision-making (15, 36, 40).

**Table 5:** Summary results on associations between reproductive coercion and women's health outcomes.

Health outcome	Findings on associations with reproductive coercion
Contraceptive use	Reduced modern contraceptive use among women who have experienced RC; findings on specific contraceptive methods vary (results inconsistent); Increased covert contraception; Lower contraceptive self-efficacy.
Unintended pregnancy	RC linked to higher odds of unintended pregnancy (results inconsistent).
Sexual health	Higher risk of STI/STDs and more frequent STI testing.
Mental and behavioral health	Increased risks of mental health concerns, including depression, anxiety, PTSD, and traumatic brain injury.

Studies examining the relationships between reproductive coercion and unintended pregnancy indicate women who had experienced reproductive coercion are more likely to have unintended pregnancies in the U.S. and India, with some studies reporting adjusted odds ratios as high as 5.39 (10–12, 18, 35, 36, 45, 57). Women experiencing reproductive coercion are also more likely to report lower pregnancy planning scores (57). The associations between reproductive coercion and unintended pregnancy aren't consistent across all studies. One U.S. study found the association attenuated to nonsignificance after adjustment for sociodemographic factors (53), Miller and colleagues found it held only among women exposed to IPV (80), and evidence on pregnancy testing was mixed (11, 34, 50). Unintended pregnancies due to reproductive coercion can lead to additional health consequences, including complications from miscarriage and pregnancy, potential for fetal anomalies resulting in increased healthcare needs, and heightened risks to maternal health (81). The association between reproductive coercion and abortion or miscarriage, however, is not as clearly defined. Research from the U.S. and South Africa suggests that women who have experienced reproductive coercion are more likely to report abortion (OR = 2.46) and are more susceptible to abuse-induced miscarriages when contraception is forcibly withheld (OR = 28.70) (12, 44, 82). Furthermore, failed abortion attempts may result in stigma and involuntary motherhood (83). Victims of reproductive coercion also face an increased risk of premature birth (OR = 8.340) and low-birth-weight neonates at term in the U.S. and suffer from higher prenatal distress in Liberia and diminished parenting competency in the U.S (33, 52, 82, 84).

Studies has also investigated the associations between reproductive coercion and sexual health outcomes, including sexually transmitted infection (STI)/sexually transmitted disease (STD) and HIV, in the U.S., South Africa, and Australia. Findings predominantly show that reproductive coercion is associated with a higher risk of STI/STDs and more frequent STI testing, with odds ratios reaching 2.7 in the U.S (11, 30, 36, 40, 43, 44, 70). In South Africa, women facing reproductive coercion have increased reported rates of HIV (aOR = 1.42), while in the U.S., these women are more concerned about HIV infection (aOR = 1.82) and show greater acceptability of PrEP, an effective HIV biomedical HIV prevention method (40, 44, 76). Only two studies found no significant associations between disclosing retrodictive coercion and STI outcomes or STI-related healthcare engagement (34, 50). The current body of research extends beyond sexual and reproductive health to explore the impact of reproductive coercion on mental and behavioral health. A significant positive association has been found between reproductive coercion and various mental health concerns, including depression, anxiety, PTSD, and traumatic brain injury, across studies in the U.S., Australia, and Côte d'Ivoire (17, 33, 40, 42, 43, 56, 61, 66, 74, 85, 86). Affected women also report heightened perceptions of future violence, increased alcohol use, and higher school absenteeism among college student populations (55, 56, 61). Only one study did not report a significant association between reproductive coercion and mental health issues (50).

### 3.7 Women's resistance strategies and reproductive coercion interventions

In the United States and Nicaragua, women experiencing reproductive coercion have adopted a broad spectrum of strategies for resistance, coping, or harm reduction. These strategies encompass a range from passive acceptance and rationalization of reproductive coercion to active resistance. Measures include sterilization, contemplation of adoption, confrontation or deception of the partner, covert use of contraceptives, reliance on spiritual faith, use of emergency contraceptives, deception and stalling, seek help from community service or family members, abortion and ending the relationship (31, 57, 87, 88). Among these, covert contraceptive use seems to be the most common strategy. However, research in Nicaragua suggests that such strategies rarely afford women autonomy over their reproductive choices over a longer time span (31). Further research focusing on marginalized adolescent girls in Mexico reveals a concerning gap: none reported awareness or utilization of any strategies to counteract reproductive coercion (89). This underscores a critical lack of capacity and self-efficacy among the most vulnerable women and girls to resist reproductive coercion, even in the short term. Women expect healthcare providers to respond proactively, including acknowledging the seriousness of reproductive abuse, asking about concurrent violence, and supporting their contraceptive needs and reproductive autonomy, including during vulnerable transitions to motherhood (29, 90); both providers and patients in the United States view integrating IPV and reproductive-coercion education into reproductive health visits favorably (91).

Interventions addressing reproductive coercion have predominantly focused on screening, education, provision of contraception, and referrals within healthcare settings. The clinical approach to reproductive coercion intervention, first proposed by Miller and colleagues in 2010, advocates for healthcare providers to recognize male partner reproductive coercion as a potential contributor to contraceptive nonadherence (80). This approach challenges the traditional attribution of nonadherence to individual factors such as lack of knowledge or ambivalence. Further, it is recommended that women be informed about reproductive coercion, its health consequences, and pregnancy risk, as well as harm reduction strategies, including the use of IUDs and injectable contraceptives (80). Early evaluations were promising, with a 71% reduction in pregnancy coercion among intervention-clinic patients and a greater likelihood of leaving unsafe relationships regardless of IPV status (92). Subsequently, the Addressing Reproductive Coercion in HEalth Settings (ARCHES) intervention was developed to enhance women's autonomy in family planning and pregnancy decisions (9, 93). ARCHES, delivered through family planning provider discussions and safety cards with health impact quizzes, harm reduction tips, and national violence-related resources, aims to support all clients, irrespective of their disclosure of reproductive coercion (9, 93). ARCHES promotes universal education and resource provision during family planning clinic visits as an alternative to traditional screening methods dependent on patient disclosure of IPV. Implemented through Project Connect, ARCHES integrates principles of trauma-informed care into provider training and interventions (93). These principles emphasize recognizing the effects of violence on individuals, fostering empowerment, and ensuring safety and nonjudgmental support in care provision (93). Evaluations of ARCHES in diverse settings, including California, Pennsylvania, and Kenya, have yielded largely positive results (91, 93–96). Patients welcome trauma-informed discussions about relationship health, and those receiving violence-related discussions or safety resources report increased confidence in their providers' concern for their safety. ARCHES also boosted providers' confidence in addressing IPV and reproductive coercion. Despite this, a 2016 study noted no significant impact on the reduction of reproductive coercion, but an increase in participants' awareness of partner violence resources and their confidence in applying harm reduction techniques (9). Efforts to enhance ARCHES have been made, for example, through the introduction of Trauma-Informed Personalized Scripts (TIPS). However, studies have indicated that adding patient messages to provider scripts (TIPS) or enhancing IPV/RC communication skills through workshops that include simulated patient interactions, did not show added benefits (94, 95). This suggests that the basic ARCHES intervention could be effective in reducing reproductive coercion and enhancing contraceptive uptake without the need for these supplementary components.

In the landscape of interventions in healthcare settings for reproductive coercion and IPV, universal education, assessment, provision of contraception and the integration of reproductive coercion screening within IPV tools are emphasized due to the co-occurring nature of reproductive coercion and IPV (31, 50, 64, 74, 97, 98). Such interventions are advocated across various healthcare settings, notably among specialists such as sexual and reproductive health clinicians, gynecology and obstetrics, cancer and gynecology specialists, abortion providers and STI treatment facilities, fertility specialists, emergency departments and maternal and neonatal health settings or domestic violence workers (50, 87, 99, 100). Not all recent studies emphasize on universal education and resource provision; some still emphasizes the importance of reproductive coercion screening. For instance, the screening program at Sexual Health Quarters (SHQ) in Australia, which is well-regarded by both patients and clinical staff, found that 61% of women who screened positive for IPV/RC were pre-existing patients, indicating missed opportunities for detection prior to the intervention (99). Implementing clinical based reproductive coercion interventions is not without challenges, which may include time constraints, the absence of routine intervention prompts, inadequate training, organizational support, and clearly defined referral pathways to domestic violence and counseling services (64, 91, 101–104). Moreover, the lack of comprehensive, evidence-based guidelines for managing reproductive coercion poses a hurdle (105). To address these challenges, researchers recommend strategies such as enhancing initial screening emphasis, specialized and follow-up training for providers, and fostering a confidential and safe environment for patient disclosures (102–104, 106, 107). The reviewed intervention studies recommend that clinicians provide non-judgmental, empathetic care with access to resources and referrals (64, 74, 98, 102). It is crucial to employ culturally sensitive and tailored screening approaches, deliver person-centered, trauma-informed care, and provide patients with harm reduction strategies, information on female-controlled contraceptives, STI testing, and resources for violence support (64, 74, 98, 102). These studies further call for providers to be equipped to support women in defining reproductive intentions and navigating partner-imposed barriers, ensure the availability and affordability of a wide range of contraceptives, and guarantee privacy during consultations to encourage open dialogue (64, 102, 106, 107).

A whole-organization approach involving efficient time management, reflective practice, and multidisciplinary collaboration is necessary to address the comprehensive needs arising from reproductive coercion (105). The lack of referral options, especially in rural areas, impedes comprehensive care, highlighting the need for improved connections between primary care and specialized support services (64, 104, 105). Training clinicians to recognize and manage reproductive coercion, alongside establishing clear referral pathways, is imperative to enhance the support for women affected by reproductive coercion. The successful implementation of clinical-based reproductive coercion interventions extends beyond healthcare providers to encompass the entire healthcare management system. System-level changes, such as integrating electronic health record prompts and linking IPV assessments to quality metrics, could significantly improve the adoption of these practices (91). Additionally, measures such as extending clinic hours, providing long-acting reversible contraception (LARC) services at accessible locations with options for same-day placement, keeping non-LARC methods readily available and removal of administrative and financial obstacles are recommended (97). To cultivate a culture of reproductive coercion and IPV screening, it is critical to involve providers at every level in the development and evaluation of protocols, aligning with their professional independence and workload management (108).

### 3.8 Beyond healthcare: addressing reproductive coercion through legislation and policy, community engagement, innovation, and support for marginalized women

To effectively address reproductive coercion, a multifaceted approach is required that extends beyond healthcare settings to tackle issues such as harmful gender and social norms. This involves challenging traditional masculine ideals centered on controlling women, engaging boys and men in community and school programs to promote rights-based approaches to safe relationships and reshape social norms around the acceptability of reproductive coercion, and committing to long-term, multi-sectoral initiatives aimed at dismantling societal norms that support male control over female reproductive rights (31, 89, 106). These initiatives aim to foster a culture where coercive behaviors are deemed unacceptable and promote respect for women's reproductive choices across different sectors. Societal factors like community silence and cultural values around motherhood, which may inadvertently normalize reproductive coercion, need to be brought to light (109). It is essential for professionals, parents, and peers to foster discussions that confront coercive behaviors and challenge misconceptions surrounding control and masculinity (110). Technological innovations like the myPlan app can support college-aged women, including those from minority groups, in safety planning and reproductive decision-making (111).

Policy-wise, there's a need for national and state leadership to train healthcare professionals in identifying and responding to IPV and reproductive coercion and to support emergency counseling resources to ensure accessible support for survivors (99). For policy reform, authors advocate framing reproductive coercion and IPV as human rights issues and push for state funding for comprehensive social support services, especially in rural areas (64). They also call for policies to improve access to abortion, particularly through telehealth, to provide timely, private, and affordable services (104). Embedding reproductive coercion in existing and new policies, such as the Australian Women's Health Strategy and the National Plan to Reduce Violence Against Women and their Children and developing a national sexual and reproductive health and rights strategy and a national healthcare professional training program that have components addressing reproductive coercion, are crucial (100). Reproductive coercion is often encompassed within legislative definitions of domestic violence due to its coercive nature, yet there's debate over its complexity in legal terms (112). Trust development is vital for disclosures of reproductive coercion, which often face credibility challenges similar to sexual assault claims in legal contexts (112). Legal analyses argue that removing legislative barriers, particularly those preventing access to contraception, is essential to achieving reproductive autonomy, even recognizing the legal system's limitations in addressing reproductive coercion (110).

Interventions should prioritize vulnerable women, such as undocumented migrants and non-native language speakers. It's important to clarify the legal risks for women seeking health interventions and ensure the availability of qualified interpreters trained in reproductive coercion and abuse (88, 113). Health professionals, police, and interpreters need specific training to distinguish reproductive coercion from other forms of violence, facilitating accurate discussions, disclosures, and appropriate referrals (113). Systematic integration of accessible interpreter services in health and policing, with an emphasis on the routine use of appropriate interpreters and the establishment of related policies, is necessary (113).

## 4 Discussion

This scoping review mapped the state of the science on reproductive coercion across 119 articles published between 2007 and 2023. Publication has grown rapidly, with most studies appearing since 2020, yet the evidence remains concentrated in high-income countries and weighted toward partner-perpetrated pregnancy coercion and birth control sabotage rather than abortion coercion or non-partner perpetration. As awareness expands and interventions such as ARCHES are adapted to lower-income settings, future research is likely to draw increasingly on LMICs. Three priorities emerge for advancing the field, including harmonizing definition and measurement, diversifying the evidence base across settings and perpetrators, and coordinating intervention and policy responses.

The concept of reproductive coercion has evolved from its initial focus on pregnancy coercion and birth control sabotage toward a broader conception encompassing abortion coercion and control during motherhood, with recent definitions emphasizing intent to control reproductive outcomes and distinguishing behaviors that have reproductive consequences but different intentions, such as “stealthing” (5, 7, 26, 29). A further conceptual distinction warranting elaboration is that between pregnancy-promoting and pregnancy-preventing coercion: most studies have concentrated on pregnancy-promoting behaviors, and although empirical evidence that coercion dynamics differ between the two remains limited, coercive control may plausibly manifest differently across these contexts, such that overlooking pregnancy-preventing behaviors may contribute to underestimation of true prevalence (5, 27). Yet most studies continue to apply the narrower interpretation, which underscore the need for broader academic and practical consensus on the construct's scope. The Miller Scale marked a major measurement advance (7, 38), but its partner-focused items, U.S. and adolescent origins, limited coverage of abortion coercion, and absence of an intent dimension constrain its scope. Coercion perpetrated by healthcare providers, including coercion linked to HIV-related stigma documented qualitatively among women living with HIV (114), remains particularly underexplored in the quantitative literature, although recent work has begun to develop provider-based measures (67). Abortion coercion is the most consequential of these gaps. It has been documented qualitatively (24, 25, 115), but few quantitative studies have specifically measured partner pressure to terminate a pregnancy, and those that did reported a low prevalence of about 4% (116). Existing quantitative work draws largely on abortion-clinic samples, including the Turnaway Study, which by design exclude women pressured to abort but who refused and women prevented from accessing abortion, and which rely on decision-making questions that are not act-based, the standard for assessing violence against women (117). Measurement should therefore extend beyond abortion clinics to antenatal and crisis pregnancy settings. The heterogeneity of abortion laws across the countries represented in the included studies further complicates measurement, outcomes, and policy: in legally restrictive settings, coercion to continue a pregnancy may predominate and abortion-related questions may be unsafe or unanswerable, whereas coercion to terminate may be more visible where abortion is permitted, so estimates are not directly comparable across jurisdictions. Abortion coercion may also share associations with intimate partner violence that are distinct from those of pregnancy-promoting coercion, which could be an important direction for future research.

The evidence base is narrow in ways that limit generalizability. Its concentration in the United States and Australia, which together account for about 71% of studies and are largely based on small, clinic-based samples, means that prevalence estimates and correlates may not generalize to settings where reproductive coercion is least studied. Future research should prioritize LMICs, where pregnancy and abortion coercion may take distinct forms and where in-law perpetration may be more salient. Beyond geography, the literature is weighted toward interpersonal, partner-perpetrated coercion. Provider-perpetrated and structural coercion remain underexamined in quantitative research, as do perpetrators' own intentions and goals. Emerging perpetrator-focused research, such as that by Alexander and colleagues (118), could inform community- and perpetrator-focused interventions. Encouragingly, the evidence is beginning to diversify. Emerging longitudinal evidence from multiple sub-Saharan African cohorts shows that reproductive coercion shapes subsequent contraceptive dynamics over time, beginning to address the field's heavy reliance on cross-sectional designs (119), and recent reviews extend the evidence to forcibly displaced populations (120), and national population-based Australian prevalence beyond clinic samples (121). Synthesizing risk factors across community, household, couple, and individual levels (4, 49, 122), the most consistent correlates are relationship instability, partner concurrency or polygyny, and lower household wealth, whereas findings on women's age, race and ethnicity, and education are mixed and context-dependent, which reinforce the need for setting-specific evidence rather than generalized risk profiles.

Reproductive coercion overlaps substantially with intimate partner, sexual, and domestic violence, can be both a result and a precursor of them, while research also demonstrates its independent impact on reproductive health, supporting its treatment as a distinct challenge within the broader context of violence against women (10, 18). It independently shapes contraceptive use, unintended pregnancy, sexually transmitted infection and HIV risk, and mental and behavioral health, with effects that vary by sociodemographic context and IPV exposure (10, 33). These findings argue for responses that are integrated yet distinct, including embedding reproductive coercion assessment within violence against women services while ensuring it is not subsumed by them. Clinical interventions have evolved from disclosure dependent screening toward universal, trauma-informed education and resource provision, exemplified by ARCHES, which has improved patient and provider confidence even where reductions in coercion itself are inconsistent (9, 89). Sustained progress requires coordinated action beyond the clinic. System-level enablers, including provider training, clear referral pathways, electronic health record prompts, and removal of administrative and financial barriers to contraception, are needed to make interventions routine, while policy responses should improve abortion access, fund survivor support, and embed reproductive coercion within national violence and sexual and reproductive health strategies (27, 104, 112). Reaching marginalized groups, including undocumented migrants and women with limited host language proficiency, additionally requires legal clarity and trained interpreters. Across all levels, addressing harmful gender norms through community and male engagement programs is essential to prevention rather than only response.

This review's strengths include its breadth across 119 studies and diverse global settings, its explicit global and life course scope covering pregnancy and abortion coercion, and its synthesis of correlates and outcomes across socio ecological levels, illustrated in the conceptual framework in Figure 2. To our knowledge, this is among the first reviews to bring these elements together. Relative to a recent scoping review of reproductive coercion and its associated health consequences (123), the present review is distinguished by its global and life course scope, its sustained attention to abortion coercion alongside pregnancy coercion, and its synthesis of multi-level correlates extending beyond the interpersonal level, although these contributions, particularly the conceptual breadth and abortion-coercion coverage that draw on qualitative evidence, should be interpreted in light of the search limitations described below.

Several limitations should be noted. The most consequential concerns the comprehensiveness of the search for qualitative evidence. The database search strings excluded records indexed with the term “qualitative”, reflecting the original protocol's primarily quantitative focus. The eligibility criteria were subsequently broadened during the review to include qualitative and mixed-methods studies, but the database searches were not re-run without this filter. The 31 qualitative studies included here were therefore identified through routes that were not subject to the filter, together with database records that did not carry the term “qualitative” in their titles, abstracts, or indexing. The extent of any resulting omission cannot be quantified. The studies most at risk of systematic omission are qualitative studies explicitly indexed with the term “qualitative” that were not captured by our citation networks or complementary sources. Conclusions concerning conceptual breadth and abortion coercion should therefore be interpreted with this limitation in mind.

Further limitations include: data extraction was performed by a single reviewer, although a two-reviewer approach was used for study selection and a standardized form enhanced accuracy; the review was restricted to English-language publications, which may have excluded relevant non-English evidence; humanitarian contexts and specialized populations such as incarcerated women were excluded by design, limiting transferability to these groups; the evidence base relies heavily on cross-sectional designs, constraining causal inference, although recent longitudinal cohorts are beginning to address this (119); and, consistent with scoping review methodology, we did not conduct a formal critical appraisal of study quality. The intentionally broad research questions, while limiting depth in any single subdomain, enabled a comprehensive overview of the field. Because the systematic search closed in 2023, the review does not systematically capture literature published in 2024–2026. A non-systematic scan of this period suggests that recent work has largely reinforced, rather than overturned, our conclusions. A fully updated systematic search remains an important direction for future work.

In conclusion, the review on reproductive coercion underscores an urgent need for a consensus on its definition and measurement, as well as comprehensive interventions that consider known risk factors and potential adverse health outcomes. Despite the significant strides made since the first article on this topic was published in 2007, the field continues to grapple with varying interpretations of reproductive coercion, largely centered on pregnancy coercion and birth control sabotage. To move forward, it is imperative to establish a more widely accepted framework that captures the full spectrum of reproductive coercion, supported by multi-sectoral interventions involving legal, educational, and social services to create a holistic support system for affected women. Finally, because the evidence base in this area is primarily based on small, homogenous samples drawn from clinical populations in high-income contexts, future research could consider prioritizing expanding its geographical scope and diversifying its participant base, ensuring a more inclusive and comprehensive understanding of reproductive coercion globally.
