# Supplementary material for: Advancing the science on measurement, determinants, and outcomes of pregnancy and abortion coercion in global settings: a scoping review
Source: Front Glob Womens Health. 2026 Oct 6;7:1854104. doi: 10.3389/fgwh.2026.1854104 (PMC13682352; doi:10.3389/fgwh.2026.1854104)
Supplement: Supplementary file 1 [file Datasheet1.docx]

**Supplementary file 1: Search strategy**

**MEDLINE through PubMed**

n=3,333 (April 2, 2023)

| **#** | **Searches** | **Results** |
| --- | --- | --- |
| 1 | (((((((reproduct* coerc*[Text Word]) OR (pregnan* coerc*[Text Word])) OR (abort* coerc*[Text Word])) OR (birth control sabotage[Text Word])) OR (contracept* sabotage[Text Word])) OR (reproduct* autonomy[Text Word])) OR (reproduct* abuse[Text Word])) OR (reproduct* violence[Text Word]) | 10,242 |
| 2 | (((women[Title/Abstract]) OR (woman[Title/Abstract])) OR (girl[Title/Abstract])) OR (girls[Title/Abstract]) | 1,534,505 |
| 3 | #2 NOT (qualitative[Title/Abstract]) | 1,500,419 |
| 4 | #1 AND #3 | 4,426 |
| 5 | (((#4) AND (English[Language])) AND (("1995/01/01"[Date - Completion] : "3000"[Date - Completion]))) AND (MEDLINE[Filter]) | 3,333 |

**Scopus**

n=1,165 (April 2, 2023)

(reproduct* coerc* OR pregnan* coerc* OR abort* coerc* OR birth control sabotage OR contracept* sabotage OR reproduct* autonomy OR reproduct* abuse OR reproduct* violence) AND (wom*n OR girl OR girls) AND NOT qualitative AND ( LIMIT-TO ( PUBYEAR,2023) OR LIMIT-TO ( PUBYEAR,2022) OR LIMIT-TO ( PUBYEAR,2021) OR LIMIT-TO ( PUBYEAR,2020) OR LIMIT-TO ( PUBYEAR,2019) OR LIMIT-TO ( PUBYEAR,2018) OR LIMIT-TO ( PUBYEAR,2017) OR LIMIT-TO ( PUBYEAR,2016) OR LIMIT-TO ( PUBYEAR,2015) OR LIMIT-TO ( PUBYEAR,2014) OR LIMIT-TO ( PUBYEAR,2013) OR LIMIT-TO ( PUBYEAR,2012) OR LIMIT-TO ( PUBYEAR,2011) OR LIMIT-TO ( PUBYEAR,2010) OR LIMIT-TO ( PUBYEAR,2009) OR LIMIT-TO ( PUBYEAR,2008) OR LIMIT-TO ( PUBYEAR,2007) OR LIMIT-TO ( PUBYEAR,2006) OR LIMIT-TO ( PUBYEAR,2005) OR LIMIT-TO ( PUBYEAR,2004) OR LIMIT-TO ( PUBYEAR,2003) OR LIMIT-TO ( PUBYEAR,2002) OR LIMIT-TO ( PUBYEAR,2001) OR LIMIT-TO ( PUBYEAR,2000) OR LIMIT-TO ( PUBYEAR,1999) OR LIMIT-TO ( PUBYEAR,1998) OR LIMIT-TO ( PUBYEAR,1997) OR LIMIT-TO ( PUBYEAR,1996) OR LIMIT-TO ( PUBYEAR,1995) ) AND ( LIMIT-TO ( LANGUAGE,"English" ) )

**CINAHL through EBSCOhost**

n=477 (April 2, 2023)

AB reproduct* coerc* OR AB pregnan* coerc* OR AB abort* coerc* OR AB birth control sabotage OR AB contracept* sabotage OR AB reproduct* autonomy OR AB reproduct* abuse OR AB reproduct* violence AND AB wom*n NOT AB qualitative

Limiters - Published Date: 19950101-; English Language

Search modes - Boolean/Phrase

**Embase**

n=3,230 (April 2, 2023)

(((((((reproduct* AND coerc* OR pregnan*) AND coerc* OR abort*) AND coerc* OR 'birth'/exp OR birth) AND ('control'/exp OR control) AND sabotage OR contracept*) AND sabotage OR reproduct*) AND ('autonomy'/exp OR autonomy) OR reproduct*) AND ('abuse'/exp OR abuse) OR reproduct*) AND ('violence'/exp OR violence) AND (wom*n OR 'girl'/exp OR girl OR girls) NOT ('qualitative'/exp OR qualitative) AND [english]/lim AND [embase]/lim AND [1995-2023]/py

*Note: The string above is reproduced as recorded in the Embase search history. Embase automatically rewrites multi-word phrases entered without quotation marks (mapping words to Emtree terms and combining them with AND), so the recorded string differs from the intended phrase-based logic used in the other databases; the implications are discussed in the manuscript’s Limitations.*

**Cochrane Library**

n=5 Cochrane Reviews; n= 211 Trials (April 1, 2023)

reproduct* coerc* in Title Abstract Keyword OR pregnan* coerc* in Title Abstract Keyword OR reproduct* abuse in Title Abstract Keyword OR birth control sabotage in Title Abstract Keyword OR reproduct* autonomy in Title Abstract Keyword - with Cochrane Library publication date from Jan 1995 to present (Word variations have been searched)

In total, the five database searches conducted on April 1-2, 2023 yielded 8,421 records (MEDLINE through PubMed: 3,333; Scopus: 1,165; CINAHL: 477; Embase: 3,230; Cochrane Library: 216), and complementary searches (Google Scholar, government and organization websites, grey literature, and citation searching) contributed a further 89 records, for a total of 8,510 records identified in the original search, as reported in the PRISMA flow diagram (Figure 1); 5 additional records were identified between May and September 2023.
