# Supplementary material for: Advancing the science on measurement, determinants, and outcomes of pregnancy and abortion coercion in global settings: a scoping review
Source: Front Glob Womens Health. 2026 Oct 6;7:1854104. doi: 10.3389/fgwh.2026.1854104 (PMC13682352; doi:10.3389/fgwh.2026.1854104)
Supplement: Supplementary file 2 [file Datasheet2.docx]

**Supplementary file 2: Result tables**

Table A1: Results on Reproductive Coercion Conceptualization in Select Studies.

| First author and year | Country of study | Type of reproductive coercion | Level of reproductive coercion | Key domains | Findings on reproductive coercion conceptualization |
| --- | --- | --- | --- | --- | --- |
| Miller 2010 | US | Pregnancy coercion; Birth control sabotage | Interpersonal (partner) | - Pregnancy Coercion - Contraceptive Sabotage | Any deliberate attempt to control a woman’s reproductive choices or interfere with her reproductive autonomy |
| Moore 2010 | US | Pregnancy coercion; Birth control sabotage; Abortion coercion; Reproductive coercion | Interpersonal (partner) | - Pregnancy promotion - Contraceptive sabotage - Sexual violence - Condom manipulation - Controlling pregnancy outcome - Interfering with health care | Coercive behaviors as qualitatively identified include specific acts of pregnancy promotion, contraceptive sabotage, sexual violence, condom manipulation, controlling pregnancy outcome, and interfering with health care. |
| Grace 2018 | US | Reproductive coercion | Interpersonal (partner) | - Contraceptive sabotage - Coercion or pressure to become pregnant - Controlling pregnancy outcomes (e.g., pressure to continue or terminate a pregnancy) | Behavior that interferes with the autonomous decision-making of a woman, with regard to reproductive health. |
| Tarzia 2021 | Not applicable | Reproductive coercion | Interpersonal (partner) | - Physical, psychological, and sexual tactics - Occurs in a context of fear and/or control in an interpersonal relationship - Centered around intent, fear and control | Reproductive coercion and abuse (RCA) is defined as any deliberate attempt to dictate a woman’s reproductive choices or interfere with her reproductive autonomy.  Issues such as structural inequalities, forced fatherhood, stealthing, and female genital mutilation should not be considered as RC. IPV and SV are the mechanisms through which RCA is perpetrated. |
| Tarzia 2020 | Australia | Birth control sabotage | Interpersonal (partner) | - “Stealthing” (non-consensual condom removal) is not qualified | Stealthing is primarily a form of sexual violence, as it lacks the specific reproductive intent that characterizes RCA. |
| Sheeran 2022 | Australia | Pregnancy coercion; Birth control sabotage | Interpersonal (partner) | - Pregnancy promotion - Pregnancy preventing | Much of the previous research focuses on forced pregnancy and condom sabotage, which effectively excludes half of those experiencing RCA and makes prevalence appear lower. |
| Pike 2022 | Not applicable | Abortion coercion; Reproductive coercion | Macrosocial; Community; Interpersonal (provider); Interpersonal (partner) | - Abortion coercion | Nature and extent of RC and in particular coerced abortion, which has been neglected in RC literature to some extent. |
| Cannon 2021 | US | Contraceptive coercion | Interpersonal (provider) | - Impact provider attitudes on women’s perceptions of contraceptive coercion | Identified clinician attitudes that may lead to patients’ perceptions of contraceptive coercion. |
| Cox 2012 | England and Wales | Reproductive autonomy | Macrosocial | - Institutionalization - Sterilization - Long-acting contraception - Permanent adoption | Past and present tactics used by the state in its attempts to limit that autonomy. |

Table A2: Sample and Prevalence of Types of Reproductive Coercion in Select Quantitative Studies.

| First author and year | Country of study | Type of study | Characteristics of study participants | Sampling and sample size | Measurement | Level of reproductive coercion | Prevalence of Reproductive Coercion (per cent) | Prevalence of Pregnancy Coercion (per cent) | Prevalence of Birth Control Sabotage (per cent) | Prevalence of Abortion Coercion (per cent) |
| --- | --- | --- | --- | --- | --- | --- | --- | --- | --- | --- |
| Miller 2010 | US | Quant; Cross-sectional | Women aged 16-29 years, English and Spanish speaking, seeking care in family planning clinics | Clinic-based; n=1319 | Miller et al. (2010); 11 items "Has someone you were dating or going out with ever: (1) told you not to use any birth control (like the pill, shot, ring, etc.)?; (2) said he would leave you if you did not get pregnant?; (3) told you he would have a baby with someone else if you didn't get pregnant?; (4) hurt you physically because you did not agree to get pregnant? and (5) tried to force or pressure you to become pregnant? (6) Have you ever hidden birth control from a sexual partner because you were afraid he would get upset with you for using it? and Has someone you were dating or going out with ever (7) taken off the condom while you were having sex so that you would get pregnant?; (8) put holes in the condom so you would get pregnant?; (9) broken a condom on purpose while you were having sex so you would get pregnant?; (10) taken your birth control (like pills) away from you or kept you from going to the clinic to get birth control so that you would get pregnant? or (11) made you have sex without a condom so you would get pregnant?"; Lifetime | Interpersonal (partner) | * | 19 | 15 | - |
| Brenner 2021 | Nicaragua | Mixed methods; Cross-sectional | Women aged 18-35 years, care-seeking in urban primary health care centers | Clinic-based; n=384 | Same to Miller et al. (2010); Lifetime and past 12 months | Interpersonal (partner) | 17.4 (lifetime); 12.3 (past 12 months) | 12.6 (lifetime); 7.4 (past 12 months) | 11.8 (lifetime); 8.7 (past 12 months) | - |
| Clark 2014 | US | Quant; Cross-sectional | Women aged 18-44 years, care-seeking and English speaking. | Clinic-based; n=737 | Same to Miller et al. (2010); Lifetime | Interpersonal (partner) | 16 | 11 | 9 | - |
| Falb 2014 | Côte d'Ivoire | Quant; Cross-sectional | Women aged 18 years and over, no history of microfinance | Community-based; n=1273 | Same to Miller et al. (2010); Lifetime | Interpersonal (partner) | 18.6 | * | * | - |
| Fay 2020 | US | Quant; Cross-sectional | Women aged 18 years and over, English or Spanish speaking, with at least one pregnancy delivered in the second trimester or later | Clinic-based; n=202 | Same to Miller et al. (2010) | Interpersonal (partner) | 8.6 | * | * | - |
| Hill 2019 | US | Quant; Cross-sectional | Female high school students who reported ever having sex with a male partner | School-based; n=550 | Same to Miller et al. (2010); Past 3 months | Interpersonal (partner) | 12 | * | * | - |
| Holliday 2017 | US | Quant; Cross-sectional | Women aged 16-29 years, English or Spanish speaking, seeking services at family planning clinics | Clinic-based; n=1234 | Same to Miller et al. (2010); Lifetime | Interpersonal (partner) | 25.9 | * | * | - |
| Jones 2016 | US | Quant; Cross-sectional | Women aged 16-29 years, seeking care, with a history of heterosexual intercourse | Clinic-based; n=2228 | Same to Miller et al. (2010); Past 3 months | Interpersonal (partner) | 7 (adolescents); 6 (young adults) | * | * | - |
| Kazmerski 2015 | US | Quant; Cross-sectional | Women aged 16-29 years, English and Spanish speaking, seeking care in family planning clinics | Clinic-based; n=1262 | Same to Miller et al. (2010); Past 3 months | Interpersonal (partner) | 13.5 | * | * | - |
| Muñoz 2023 | US | Quant; Cross-sectional | Students in public high schools | School-based; n=370 | Same to Miller et al. (2010); Lifetime | Interpersonal (partner) | 11.4 | 10 | 3.3 | - |
| Northridge 2017 | US | Quant; Cross-sectional | Girls aged 14-17 years that are sexually active | Clinic-based; n=149 | Same to Miller et al. (2010); Lifetime | Interpersonal (partner) | 19 | * | * | - |
| Sutherland 2015 | US | Quant; Cross-sectional | College women aged 18-25 years, enrolled either full- or part-time, English speaking, and screened positive for relationship | School-based; n=972 | Same to Miller et al. (2010); Lifetime | Interpersonal (partner) | 8 | 6.8 | 3.9 | - |
| McCauley 2017 | US | Quant; Cross-sectional | Women aged 16-29 years, seeking care in family planning clinics | Clinic-based; n=4674 | Adapted shorter form; "In the past 3 months, has someone you were dating or going out with: (1) Told you not to use any birth control (like the pill, shot, ring, etc.); (2) Taken your birth control (like pills) away from you or kept you from going to the clinic to get birth control; (3) Made you have sex without a condom so you would get pregnant; (4) Taken off the condom while you were having sex, so you would get pregnant; (5) Put holes in the condom or broken the condom on purpose so you would get pregnant."; Past 3 months | Interpersonal (partner) | 6.3 | * | * | - |
| Campbell 2023 | US | Quant; Cross-sectional | Black women aged 18-44 years, care-seeking at public health clinics, with high-risk sexual behavior | Clinic-based; n=305 | Adapted shorter form; McCauley et al. (2017); Lifetime and past 12 months | Interpersonal (partner) | 27.2 (lifetime); 12.1 (past 12-month) | 19.5 (lifetime); 9.1 (past 12-month) | 15.8 (lifetime); 5.4 (past 12-month) | - |
| Capasso 2019 | US | Quant; Experimental | African American women aged 17-24 years, with a history of alcohol consumption and high-risk sexual behavior | Community-based; n=560 | Adapted shorter form; select 4 items from Miller et al. (2010); Past 3 months | Interpersonal (partner) | 22.9 (lifetime); 14.5 (past 3 months) | * | * | - |
| Dozier 2022 | Ethiopia | Quant; Cross-sectional | Women aged 15-49 years | Internet-based; n=2469 | Adapted shorter form; McCauley et al. (2017); Past 12 months | Interpersonal (partner) | - | 17.7 | - | - |
| Grace 2022 | US | Quant; Cross-sectional | Women aged 18 years or older, English speaking, with a history of intimate partner violence and receiving housing support services | Service-based; n=70 | Adapted shorter form; McCauley et al. (2017); Past 3 months | Interpersonal (partner) | 16.4 | * | * | - |
| Kraft 2021 | US | Quant; Cross-sectional | Young women aged 14-19 years, self-identified as African American or black, had vaginal sex with a male partner recently, were not pregnant and wanted to avoid pregnancy for at least 12 months | Clinic-based; n=741 | Adapted shorter form; McCauley et al. (2017); Lifetime | Interpersonal (partner) | 20 | * | * | - |
| McCloskey 2020 | South Africa | Quant; Cross-sectional | Women aged 15-29 years, understood any of four languages (English, SeSotho‐Setswana, isiZulu, or Shona), sexually active, and were willing to share HIV results | Clinic-based; n=882 | Adapted shorter form; three items from McCauley et al. (2017); Lifetime | Interpersonal (partner) | - | 40.8 | - | - |
| PettyJohn 2021 | US | Quant; Cross-sectional | Adolescent women aged 16-24 years, seeking services from youth-serving agencies affiliated with a child welfare system | Service-based; n=136 | Adapted shorter form; McCauley et al. (2017); Lifetime | Interpersonal (partner) | 30.1 | * | * | - |
| Phillips 2016 | US | Quant; Cross-sectional | Women aged 18-45 years, English or Spanish speaking, able to get pregnant, and sexually active with a man in the past year | Clinic-based; n=97 | Adapted shorter form; "Has a sexual partner (1) ever told you not to use birth control? (2) ever said he would leave you if you did not get pregnant? (3) ever taken off the condom or broken a condom on purpose while you were having sex so that you would get pregnant? (4) ever taken your birth control (such as pills) or prevented you from going to the clinic to get birth control, so that you would get pregnant? (5) ever made you have sex without a condom so that you would get pregnant?"; Lifetime | Interpersonal (partner) | 24 | * | * | - |
| Swan 2021 | US | Quant; Cross-sectional | Self-identifed as a cis-gender or transgender person assigned female at birth aged 18-49 years | Internet-based; n=628 | Adapted shorter form; McCauley et al. (2017); Past 12 months | Interpersonal (partner) | 15.6 (sample 1); 16.4 (sample 2) | * | * | - |
| McCauley 2014 | Côte d’Ivoire | Quant; Cross-sectional | Women aged 18 years or older | Community-based; n=953 | Adapted to local context; added in-law abuse items from Hossain et al. (2010) and Raj et al. (2011); Lifetime | Interpersonal (family); Interpersonal (partner) | 18.5 (partner-perpetrated); 5.5 (in-law-perpetrated) | * | * | - |
| Silverman 2019 | India | Quant; Cross-sectional | Women aged 15-49 years who were married | Population-based; n=1770 | Adapted to local context; 8 items related to women’s experiences of coercion or force from husbands or in-laws regarding reproductive choices, including use of family planning methods, based on McCauley et al. (2017) and Miller et al. (2014) and adapted for the Indian context; Lifetime | Interpersonal (family); Interpersonal (partner) | 12 | * | * | - |
| Silverman 2020 | Niger | Quant; Cross-sectional | Girls aged 13-19 years, married, fluent in Hausa or Zarma, and currently sterilized | Community-based; n=1072 | Adapted to local context; 6 items regarding experiences of male partners’ interference with FP method use, including pregnancy pressure and contraceptive sabotage from McCauley et al. (2017) that was adapted to the Niger context based on formative data and stakeholder input. Participants were asked whether their husbands had ever either "(1) tried to force or pressure them to become pregnant, (2) taken their FP method away from them, (3) kept them from going to the clinic to access FP methods, (4) said they would leave if they did not get pregnant, (5) hurt them physically because they did not get pregnant and (6) if anyone including their husbands, in-laws, or co-wives pressured them, made them feel badly, or treated them badly in the past 12 months for not having a child."; Lifetime and past 12 months | Interpersonal (partner) | - | 10.2 | - | - |
| Tomar 2020 | India | Quant; Cross-sectional | Women aged 15-49 years, currently married | Population-based; n=1424 | Adapted to local context; 8 items relating to coercion or force used by the husband or in-laws to limit women’s reproductive autonomy: whether woman’s husband or in-laws ever (1) stopped her from going or refused to give permission for her to go to a clinic or community health event to get family planning; (2) destroyed, hidden, or taken a family planning method (such as pills) away from her; (3) told her that they would abandon her if she tried to prevent or delay getting pregnant; (4) told her that she would be beaten if she tried to prevent or delay getting pregnant; (5) told her that it was against their religion or culture to use family planning; (6) told her that women who use family planning do this so that they can have sex with other men; (7) told her that she could not use family planning because she did not have any or enough sons; (8) or not permitted her to use contraceptives; Lifetime | Interpersonal (family); Interpersonal (partner) | 15.1 | * | * | - |
| Wood 2022 | Ethiopia | Quant; Cross-sectional | Women aged 15-49 years who were married or living with a partner at time of interview | Population-based; n=5542 | Adapted to local context; 5 items pregnancy coercion sub-scale from McCauley et al. (2017) and Miller et al. (2010). Items from the condom manipulation sub-scale were not included given low prevalence of condom use within ongoing partnerships in Ethiopia; Past 12 months | Interpersonal (partner) | - | 20 | - | - |
| Wood 2023 | Burkina Faso, Côte d’Ivoire, Democratic Republic of Congo (DRC), Kenya, Nigeria, Niger, India, Uganda | Quant; Cross-sectional | Women aged 15-49 years, married or cohabitating and in need of contraception | Population-based; n=Burkina Faso n=2767; Côte d’Ivoire n=1561; Kongo Central, Democratic Republic of Congo (DRC) n=830; Kinshasa, DRC n=846; Kenya n=4588; Kano, Nigeria n=535; Lagos, Nigeria n=612; Niger n=1525; Rajasthan, India n=3017; Uganda n=2020 | Adapted to local context; 6 items from the pregnancy coercion subscale from McCauley et al. (2017) and Miller et al. (2010); Past 12 months | Interpersonal (partner) | - | 7.1 (Burkina Faso); 6.2 (Côte d’Ivoire); 20.3 (Kongo Central, DRC); 11.9 (Kinshasa, DRC); 7 (Kenya); 5.7 (Kano, Nigeria); 5 (Lagos, Nigeria); 3.1 (Niger); 3.9 (Rajasthan, India); 16.9 (Uganda) | - | - |
| Cheng 2021 | Australia | Quant; Cross-sectional | Women aged 16 years and over, care seeking | Clinic-based; n=5497 | Adapted with major modifications; "Has your partner or ex-partner ever (1) interfered with your choice or ability to use contraception? (2) forced you to become pregnant (3) forced you to continue a pregnancy that was unwanted/unplanned and (4) forced you to terminate a pregnancy that you wanted to continue; Lifetime | Interpersonal (partner) | 2.3 | * | * | - |
| Decker 2021 | Abidjan, Coˆte d’Ivoire; Nairobi, Kenya; and Lagos, Nigeria. | Quant; Cross-sectional | Unmarried adolescent and young adults male and female aged 15-24 years | Snowball; Nairobi (n=332), Abidjan (n=555), and Lagos (n=179) | Adapted with major modifications; single item "has a partner ever pressured you not to use birth control, taken your birth control (like pills away from you, or kept you from going to the clinic to get birth control?”; Lifetime | Interpersonal (partner) | 19.8 (Nairobi); 13.6 (Abidjan); 28.2 (Lagos) | - | - | - |
| Fleury-Steiner 2020 | US | Quant; Cross-sectional | Women aged 18 years and over, seeking an order of protection against a male current or former intimate partner | Service-based; n=172 | Adapted with major modifications; an 8-point scale (0 = never to 7 = every day) how often their partner or ex-partner committed each of six types of reproductive coercion, including poking holes in a condom or hiding her birth control | Interpersonal (partner) | 26.7 | * | * | - |
| Grace 2022 | US | Quant; Cross-sectional | Female college students aged 18-24 years, reporting a recent history of intimate partner violence | School-based; n=354 | Adapted with major modifications; 2 items "in the last 6 months (1) has your partner tried to get you pregnant when you didn’t want to be (e.g. by telling you not to use birth control, messing with your birth control, not using condoms, breaking condoms)? and (2) have you prevented a pregnancy by using emergency contraception (Plan B, ella, Take Action, My Way, etc.) or ended a pregnancy using other methods, and did not tell your partner about it because you were afraid of your partner?"; Past 6 months | Interpersonal (partner) | 24.3 | - | - | - |
| Grace 2022 | US | Quant; Cross-sectional | Women aged 15-45 years, self-identified as Latina, Hispanic or Spanish, and had a dating or sexual partner in the past year | Clinic-based; n=482 | Adapted with major modifications; 13 items from adaptations of existing measures, adding additional questions on abortion coercion (tried to make you get an abortion; tried to stop you from getting an abortion; violence or threats to try to make you get an abortion; violence or threats of violence to try to stop you from getting an abortion) and adapted to isolate pregnancy-promoting intent from coercive behaviors; Past 12 months | Interpersonal (partner) | 16.8 | * | * | * |
| Grace 2022 | US | Mixed methods; Cross-sectional | Women aged 15-45 years, self-identified as Latina, Hispanic or Spanish, and had a dating or sexual partner in the past year | Clinic-based; 13 interviews; 482 surveys | Adapted with major modifications; 13 items from adaptations of existing measures, adding additional questions on abortion coercion (tried to make you get an abortion; tried to stop you from getting an abortion; violence or threats to try to make you get an abortion; violence or threats of violence to try to stop you from getting an abortion) and adapted to isolate pregnancy-promoting intent from coercive behaviors; Past 12 months | Interpersonal (partner) | 16.8; 9.5 (with perceived pregnancy intent) | * | 13.7 | * |
| John 2018 | Ethiopia | Quant; Cross-sectional | Ever-married women aged 18-45 years | Population-based; n=4149 | Adapted with major modifications; 4 items "in the past 12 months, has your current husband/partner (1) told you not to use any birth control? (2) hurt you physically because you did not agree to get pregnant? (3) taken your birth control away from you or kept you from going to the clinic to get birth control so that you would get pregnant? (4) Does your partner support your decision about when or if you want to become pregnant?"; Past 12 months | Interpersonal (partner) | 57.6 | - | - | - |
| Katz 2017 | US | Quant; Cross-sectional | Undergraduate female students, with a report of past consensual vaginal sex with at least one male partner | School-based; n=223 | Adapted with major modifications; 12 items adapted from Miller et al. (2010) and Moore et al. (2010). Respondents were asked to list the number of male partners with whom they experienced different forms of indirect and direct pressure or acts that could increase their risk for pregnancy. Women were asked to identify how many partners had engaged in each of the following behaviors: "(1) told you not to use any birth control (like the pill, shot, ring, etc.), (2) said he would leave you if you did not get pregnant, (3) told you he would have a baby with someone else if you didn’t get pregnant, (4) hurt you physically because you did not agree to get pregnant, (5) tried to force or pressure you to become pregnant, (6) took off the condom while you were having sex, (7) put holes in the condom that you two were going to use, (8) broke a condom on purpose while you were having sex, (9) took your birth control away from you, (10) kept you from going to the doctor’s office or clinic to get birth control, (11) made you have sex without a condom", and "(12) Have you ever hidden birth control from a sexual partner because you were afraid he would get upset with you for using it?"; Lifetime | Interpersonal (partner) | 30 | * | * | - |
| Samankasikorn 2019 | US | Quant; Cohort | Women aged 17 years and over who have recently given birth | Population-based; n=20252 | Adapted with major modifications; Participants were asked if their partners, including current or ex-husbands, “tried to keep them from using birth control and getting them pregnant when they did not want to.”; Lifetime | Interpersonal (partner) | 1.1 | - | - | - |
| Swan 2021 | US | Quant; Cross-sectional | Cisgender college female and male students endorsing at least one sexual partner in the past year | School-based; n=431 | Adapted with major modifications; 4 items assessing the frequency in the past 12 months in which a sex partner had: "(1) refused to use or kept you from using birth control or condoms when you wanted to prevent pregnancy, (2) refused to use or kept you from using condoms to prevent sexually transmitted infections (STIs), (3) accused you of wanting to use birth control or condoms so that you could have sex with other people, and (4) pressured you to have a baby by threatening to leave you or have a baby with someone else.”; Past 12 months | Interpersonal (partner) | 14 (females); 6.2 (males) | - | - | - |
| Swan 2022 | US | Quant; Cross-sectional | Undergraduate student aged 18-24 years | School-based; n=387 | Adapted with major modifications; single item conceptualized as relevant to sexual minority populations. Participants reported their experiences with reproductive coercion by indicating whether (1=yes) or not (0=no) their sexual and/or dating partner had ever refused to use the safe sex methods that they requested; Lifetime | Interpersonal (partner) | 6.3 | - | - | - |
| Thaller 2016 | US | Quant; Cross-sectional | Women seeking care at Title X reproductive health clinics | Clinic-based; practitioner screenings (n = 2,108) and anonymous surveys (n = 84) | Adapted with major modifications; 2 items “has a partner tried to get you pregnant when you did not want to be?” and “have you ever hidden birth control from a partner so he won’t get you pregnant?”; Lifetime | Interpersonal (partner) | 3.3 | - | - | - |
| Willie 2018 | Liberia | Quant; Cross-sectional | Women aged 18-30 years, receiving/received prenatal services from a local community health clinic, and between 13 and 24 weeks of gestational age | Clinic-based; n=195 | Adapted with major modifications; select items from Miller et al. (2010) and modified for the current pregnancy Willie et al. (2017b). | Interpersonal (partner) | 9 | * | * | - |
| Clark 2008 | Jordan | Quant; Cross-sectional | Literate, ever-married women | Clinic-based; n=517 | Other: Have your husband or others ever refused to use a method or tried to stop you from using a method to avoid your becoming pregnant (yes / no)? If yes, in what ways did he let you know that he disapproved of using methods to avoid your becoming pregnant: (1) told you that he did not approve; (2) shouted/became angry; (3) threatened you; (4) beat you/ physically assaulted you; (5) took or destroyed the method; Lifetime | Interpersonal (family); Interpersonal (partner) | 20 | * | * | - |
| Foster 2012 | US | Quant; Cross-sectional | Women who sought abortions at one U.S. clinic | Clinic-based; n=5109 | Other: Whether women felt pressured by others to have the abortion was assessed with the item "I’m here for an abortion mostly because someone else wants me to"; we considered a woman to have felt pressured if she replied "true" or "kind of" and had low confidence in her abortion decision. | Interpersonal (family); Interpersonal (partner) | 4 | - | - | 4 |
| Huslage 2023 | US | Quant; Cross-sectional | Self-identify as a woman or female to male transgender person aged 18-50 years | Internet-based; n=632 | Other: Participants were asked about their perceptions of contraceptive pressure in three different circumstances; however, “pressure” was not defined for participants: (1) have you ever felt pressure to become sterilized (i.e., use a permanent method to prevent becoming pregnant); (2) have you ever felt pressure to use birth control; and (3) have you ever felt pressure NOT to use birth control; Lifetime | Community (e.g., community organizations, social norms); Interpersonal (provider); Interpersonal (family); Interpersonal (partner) | 49.5 | - | - | - |
| Nemeth 2020 | US | Quant; Cross-sectional | Adolescents who reported they had been in a dating or romantic relationship within the past 12 months | School-based; n=14,927 (2010 survey) and n=11,585 (2014 survey) | Other: Adolescent Reproductive and Sexual Coercion (ARSC); 5 items that captured a range of coercive verbal and physical tactics. Participants were asked: "In the last 12 months, how many times has a current or previous boyfriend/girlfriend said to you: (1) 'you want us to use birth control or condoms so you can sleep around with other people' (UCR); (2) 'if we have a baby, you will never have to worry about me leaving you. I will always be around' (BLEVR); and (3) 'you would have a baby with me if you really loved me (BLOVR)?' (4) has a current or previous boyfriend or girlfriend not allowed you to use birth control or condoms when you wanted to (BCR); and (5) forced you to have sex when you were not using birth control or condom (BC2R)?" Response options for all items included: 0 times, 1–2 times, 3–5 times, 6 or more times, “Yes, this happened before, but not in the past 12 months,” and “Not in a dating or romantic relationship in the past 12 months.”; Past 12 months | Interpersonal (partner) | 41.8 (females); 30.6 (males) | - | - | - |
| Price 2022 | Australia | Quant; Cross-sectional | Callers seeking support in relation to an unplanned or unwanted pregnancy | Service-based; n=3117 | Other: Counselors measured the presence of reproductive coercion from a single perpetrator action from any of the three temporal domains of reproductive coercion: pregnancy pressure, contraceptive sabotage, or pregnancy outcome control | Interpersonal (partner) | 5.9 (first contact); 17.8 (second contact) | * | * | * |
| Sheeran 2022 | Australia | Quant; Cross-sectional | People contacted the counselling services, include pregnancy decision making counselling, post-abortion counselling or information about pregnancy options | Clinic-based; n=5107 | Other: Counsellor identified behaviours that were consistent with reproductive coercion. Reproductive coercion that promoted pregnancy included behaviours that had resulted in the person becoming pregnant (i.e., incessant pressure to be pregnant, psychological, or physical harm or threats if does not get pregnant or refuses sex, forced sex causing pregnancy, or contraception sabotage), as well as pressure or coercion to continue with the pregnancy. Reproductive coercion that prevented pregnancy/promoted abortion included emotional blackmail, threats, pressure, or coercion to terminate the pregnancy, or physical violence to induce miscarriage. | Interpersonal (partner) | 15.4; 6 (towards pregnancy) and 7.5 (towards pregnancy prevention or abortion) | - | - | - |

Note: "-" indicates data were not collected, and "*" denotes data were collected but not published in the study. Researchers may choose to either remove one of the original questions (Muñoz, 2023), add one or two new questions (Clark, 2014), or refer to a later article discussing the Miller scale (Brenner, 2021). For ease of categorization, these modifications are all classified as "Similar to Miller et al. (2010)".

Table A3. Results on Reproductive Coercion Risk Factors.

| First author and year | Country of study | Findings on Reproductive Coercion Risk Factors |
| --- | --- | --- |
| Brenner 2021 | Nicaragua | - Women’s education: higher education correlates with reduced RC risk. University-educated women show a 78% lower 12-month RC prevalence compared to uneducated women (APRR 0.22, 95%CI 0.07-0.67). - Women’s working status: students report an 87% lower lifetime CS prevalence than housewives (APRR 0.13, 95%CI 0.20–0.77). - Last partner’s education: women with university-educated partners report a 66% lower lifetime CS prevalence than those with uneducated partners (APRR 0.34, 95%CI 0.11–0.99). - Last partner’s working status: women with employed partners associate with a 51% lower lifetime RC prevalence compared to those with unemployed partners (APRR 0.49, 95%CI 0.27–0.88). |
| Capasso 2019 | US | - Multiple partners: women experiencing PC had 2.37 times the odds of reporting two or more sexual partners compared to women without RC (95%CI = 1.43 to 3.92). - Pregnant history: 89% (AOR = 1.89, 95% CI = 1.12 to 3.18) more likely to have ever been pregnant. - Abortion or miscarriage history: 126% (AOR = 2.26, 95% CI = 1.21 to 4.24) more likely to have had an abortion or miscarriage. |
| Clark 2008 | Jordan | - Rural: respondents who reported experiencing interference were more likely to attend a rural clinic than those without reported interference (t-test = 7.2; p = 0.01). |
| Clark 2014 | US | - Relationship status: women who reported RC were more likely to be single or in a dating relationship (P<.01); women who were single or in a dating relationship were 2 times more likely to report RC (AOR 2.16, 95%CI 1.26-3.7, P<.01), and respondents uncertain of their relationship status were almost 6 times more likely to report RC (AOR 5.57, 95%CI 1.86-16.67, P<.01). - Current pregnancy status: women who experienced RC were less likely to report currently being pregnant (P = .01); women who were currently pregnant were less likely to report RC (AOR 0.6, 95%CI 0.37-0.97, P=.04). |
| Decker 2021 | Abidjan, Coˆte d’Ivoire; Nairobi, Kenya; and Lagos, Nigeria. | - Transactional sex outside relationship: in Lagos, transactional sex outside of the relationship was associated with RC (APR:2.62, 95%CI:1.39–4.97). |
| Fay 2020 | US | - Age: women who reported RC at the time of their last pregnancy were significantly younger than their counterparts (P=.03). - Education: less likely to have completed some college (P<.01). - Health: more likely to report diagnoses of anemia (P=.04) and anxiety (P=.03). |
| Grace 2022 | US | - Race: women who experienced RC were more likely to be races other than White than women who did not experience RC (43.0% vs. 29.3%, p = 0.019). - Relationship type: more likely to be in an “on again/off again” relationship (48.2% vs. 34.2%, p = 0.022). - Age, ethnicity, living with partner, alcohol abuse, drug abuse and age difference with partner were not associated with RC. |
| Grace 2022 | US | - Birth history: participants who did not have children with their abusive partner were significantly more likely to experience RC than those who did (41.2% vs 6.1%, p=0.002). - Family size: participants who experienced RC had a significantly smaller family size (2.82 vs 4.76, t=3.29, p=0.002). |
| Grace 2022 | US | - Age: participants who experienced RC were younger than those who did not experience RC (27.60 vs. 31.06, p < .001); (AOR=0.95, 95% CI¼ [0.91, 1.00], p =.038). - Origin: more likely to be born in the United States (18.8% vs. 7.1%, p = .001). - Pregnancy status: not currently being pregnant at the time of the survey (82.7% vs. 70.8% p= .028). - Abortion history: having had one or more abortions (16.7% vs. 5.7%, p= .001). - Relationship status: significantly less likely to be married (legally or common-law; 48.1% vs. 71.9%, p < .001), and more likely to have had more than one past-year partner (23.5% vs. 8.7%, p < .001). - Partner binge drinks: RC was significantly associated with having a partner who binge drinks (48.8% vs. 24.6%, p < .001). - Partner uses drug: RC was significantly associated with having a partner who uses drugs (17.5% vs. 3.0%, p < .001). |
| Hill 2019 | US | - Race and ethnicity and year of schooling were not associated with RC. |
| Holliday 2017 | US | - Race and ethnicity: (OR)Black = 2.69, 95% CI = 1.90–3.79 and ORmultiracial = 1.88, 95%CI = 1.46–2.41] relative to White women. |
| Katz 2019 | US | - Relationship: compared with relationships without partner contraceptive interference (CI), relationship involving partner CI was characterized by significantly higher levels of conflict engagement by partners (p < .001) and higher levels of withdrawal by women (p= .001). |
| Kraft 2021 | US | - Age: young women who experienced RC (either one or two types) were older (17.28 vs. 17.62 vs. 17.48, p=0.05). - Education: more likely to have completed 12th grade (p=0.05). - Multiple partners: having two or more partners in the past 3 months (p<0.01). - Psychosocial measures: scored lower on the STD knowledge (p=0.04), future orientation (p=0.03), and perceived life chance measures (p<0.01). |
| Muñoz 2023 | US | - Race and ethnicity: Hispanic/Latinx participants had a significantly higher lifetime prevalence of RC when compared to White/Non-Hispanic participants (14.8% vs. 5.6%, χ2 (1) = 5.18, p = .023). |
| PettyJohn 2021 | US | - Use alcohol or drugs before sex: young women reporting RC had elevated odds for using alcohol or drugs before sex (AOR = 4.34, 95% CI: 1.72, 10.97). - Age difference: having sex with a male partner 5 years or more older (AOR = 7.32, 95% CI: 2.84, 18.87). - No significant differences emerged between RC and sociodemographic characteristics. |
| Phillips 2016 | US | - Race: a lifetime history of RC was associated with self-reported white race (risk ratio [RR] = 2.82, 95% CI 1.32–6.06, P = 0.01), Latina women were less likely to report RC compared with non-Latina women (RR = 0.36, 95% CI 0.15–0.84, P = 0.02). - Employment: Women who were currently employed were more likely to report RC than those who were unemployed (RR = 3.18, 95% CI 1.28–7.86, P = 0.01). - Personal safety: current lack of personal safety were associated with having experienced RC (RR = 3.51, 95% CI 1.87–6.60, P < 0.01). - Transactional sex: a history of transactional sex for money or a place to stay were associated with having experienced RC (RR = 2.36, 95% CI 1.12–4.96, P = 0.02; RR = 2.7, P = 0.02). - No association between RC and other demographic characteristics, including age, marital status, presence of a current live-in partner, level of education, income or having public insurance (all P > 0.05). |
| Petterson 2018 | United Kingdom and US | - Hostile sexism: was related to support for men having the right to prevent their pregnant partner from having an abortion, the endorsement of men’s right to withdraw financial support for the child if a woman chooses not to terminate her pregnancy, support for men’s right to veto their female partner’s decisions during pregnancy and childbirth, and the perceptions that men have the right to constrain women’s reproductive choices. |
| Price 2022 | Australia | - Age: all women who disclosed RC were most likely to be between the ages of 25 and 29 years. Relationship status: identify as separated. |
| Samankasikorn 2019 | US | - Age: women less than 30 years old were at greater risk of RC than participants 30 years and older (p < .0001). - Race: Black women were more likely to experience RC. - Education: less than a high school education (p < .0001). - Marital status: single marital status (p < .0001). - Household income: a household annual income less than $22,000 (p < .0001). |
| Sheeran 2022 | Australia | - Race: significant differences between the proportion of participants reporting coercion that promoted pregnancy who identified as Aboriginal and/or Torres Strait Islander and those who did not, χ2(1, N=5107)=7.789, p=0.005). |
| Skracic 2021 | US | - Race: Black, non-Hispanic women were more likely to report both the verbal only and behavioral types of RC (p < 0.05). - Health insurance: women with public health insurance (p < 0.008). - Age: women aged 20-29 years were most likely to report the verbal-only type, whereas women aged 30 and older were more likely to report a behavioral form of RC compared with women aged 16-19 years (p < 0.001). - Fertility intention: women who did not want a(nother) child were more likely to report both forms of RC (p = 0.001). - History of unintended pregnancy: women who had a prior unintended pregnancy were more likely to have experienced the verbal-only form of RC (p = 0.04). |
| Sutherland 2015 | US | - Race and ethnicity: Hispanic women having higher rates. |
| Swan 2021 | US | - Pregnancy fatalism: positively associated with RC in both samples (Sample 1 t(300)= -7.150, p < 0.001; Sample 2 t(299)=-7.078, p< 0.001). |
| Swan 2022 | US | - Sexual orientation: participants who reported sexual attraction to people of multiple genders were at increased risk of experiencing RC (13%) compared to those attracted to women only (3%) or men only (5%; χ2(2) = 7.57, p = .023). |
| Wood 2022 | Ethiopia | - Birth history: increasing parity was associated with decreased odds of experiencing pregnancy coercion among women with one child (aOR = 0.69; 95% CI: 0.52–0.92), two to three children (aOR = 0.49; 95% CI: 0.37–0.63), and four or more children (aOR = 0.56; 95% CI: 0.43–0.74), compared to nulliparous women. - Religion: “Other” religion, comprising primarily Catholic participants, as compared to Orthodox, was associated with increased risk of less severe pregnancy coercion (aRRR = 1.94; 95% CI = 0.99–3.80); this association was not observed for more severe pregnancy coercion. |
| Wood 2023 | Burkina Faso, Côte d’Ivoire, Democratic Republic of Congo (DRC), Kenya, Nigeria, Niger, India, Uganda | - Household wealth: increased household wealth was significantly associated with a decreased odds of past-year RC in Kinshasa (aORhighest=0.29; 95% CI=0.11–0.75; p<0.05), Kenya (aORmiddle=0.68; 95% CI=0.50–0.93; p<0.05), and Kano (aORhighest=0.17; 95% CI=0.03–0.98; p<0.05). - Relationship status: living with a partner while unmarried, as compared to married, was significantly associated with increased odds of past-year RC in Burkina Faso (aOR=2.84; 95% CI=1.70–4.75; p<0.001) and Uganda (aOR=1.86; 95% CI=1.34–2.59; p<0.001). - Polygyny: associated with an increased odds of past-year RC in Burkina Faso (aOR=1.59; 95% CI=1.00–2.27; p<0.05), Kinshasa (aOR=2.37; 95% CI=1.01–5.57; p<0.05), Kenya  (aOR=1.93; 95% CI=1.26–2.94; p<0.01), Lagos (aOR=2.78; 95% CI=1.13–6.86; p<0.05); Rajasthan (aOR=10.76; 95% CI=6.10–18.96; p<0.001), and Uganda (aOR=1.78; 95% CI=1.24–2.55; p<0.01). - Partner's education: having a partner with a secondary or higher education level was protective against RC in Kenya (aOR=0.67; 95% CI=0.49–0.96; p<0.05) and Kano, Nigeria (aOR=0.23; 95% CI=0.05, 0.96; p<0.05). - Birth history: a parity of two or higher, as compared to one or fewer births, was protective against RC in Kongo Central (aOR=0.52; 95% CI=0.30–0.90; p<0.05) and Kenya (aOR=0.59; 95% CI=0.40–0.86; p<0.01). |
| Willie 2021 | US | - Age: women who reported birth control sabotage were slightly older than women who did not report birth control sabotage (29.6 years vs. 27.9 years, p=.020). - Race and ethnicity: a higher proportion of women who reported birth control sabotage identified as non-Hispanic Black or African American women vs. women who did not report birth control sabotage (52.3% vs.32.3%, p=<.001). - Transactional sex: Women who reported birth control sabotage had a greater odds of ever engaging in exchange sex (AOR, 2.77; 95%CI, 1.17–6.53; p = .020). - Multiple partners: having multiple sexual partners in the past 6 months (AOR, 1.96; 95%CI, 1.21–3.18; p = .006). |
| Wood 2023 | Kenya | - Partner’s concurrent partnership: associated with increased RC (aIRR=1.33; 95% CI=1.18-1.53; p<0.001). - Couple communication: protective for RC (aIRRR=0.96; 95% CI=0.94-0.97; p<0.001). - Unwanted pregnancy: not wanting previous pregnancy at all (aIRR=1.29; 95% CI=1.10-1.53; p=0.009) were associated with increased RC. - Access: perceived contraception access difficulty of inconvenient use (aIRR=1.36; 95% CI=1.17-1.57; p=0.01) were associated with increased RC. - Age: in comparison to 18-20 year olds, older age groups displayed protective associations for RC incidence (aIRRR range=0.70-0.75; p=0.02). - Cohabitation with in-laws: was associated with increased incidence of pregnancy coercion (aIRR=1.79; 95% CI=1.02-3.15; p=0.05). - Religion: Muslim religion (aIRR=1.49; 95% CI=1.02-2.19; p=0.05) was associated with increased incidence of condom manipulation. - Community level gender norms, length of relationship, women's education, migration status, ethnicity, and number of pregnancies or children are not significant. |

# Table A4. Results on the Intersection of Reproductive Coercion and Intimate Partner Violence (IPV), Sexual Violence (SV) or Domestic Violence (DV).

| First author and year | Country of study | TYpe of analysis | Findings on intersection with IPV |
| --- | --- | --- | --- |
| Alexander 2021 | US | - Prevalence of RC without IPV and with IPV - Association between physical or sexual IPV (independent variable) and RC experiences (dependent variables) | - Nearly 10% of the young women reported experiencing RC without IPV; 38% of the sample reported experiencing both types of violence. - Women with lifetime physical or sexual IPV experiences were more likely to report RC (57.6% vs. 42.4%, p < .001). |
| Campbell 2023 | US | - Association between forced sex history (independent variable) and RC experiences (dependent variables) | - Women reporting forced sex were nearly three times more likely to have experienced RC in the past 12-months compared to women not reporting forced sex (AOR=2.72, 95% CI 1.27, 5.91). |
| Cheng 2021 | Australia | - Association between RC experiences (independent variables) and DV history and consultation (dependent variables) | - Women who disclosed RC were 7.94 times more likely to have DV disclosed (P < 0.0001) and were two times more likely to have discussed domestic and family violence in their consultation than women who did not disclose RC (15% vs 7.8%, respectively; P = 0.0009) after adjusting for demographics and clinic attended. |
| Clark 2008 | Jordan | - Association between physical violence, sexual violence, or controlling behaviors history (independent variables) and RC experience (dependent variable) | - Women who reported ever experiencing physical violence, sexual violence, or controlling behaviors perpetrated by their husband were 2.4 times, 3.1 times, or 1.4 times more likely also to have reported experiencing interference with their attempts to avert pregnancy. |
| Clark 2014 | US | - Co-occurrence of RC and IPV - Association between RC experiences (independent variables) and IPV (dependent variable) | - Among women reported RC, 32% (95% CI, 23-41%) also screened positive for IPV within the same relationship. - 46.6% of the women who experienced birth control sabotage also reported IPV in that same relationship (95% CI, 33.7-59.4%), and 34% (95% CI, 23-44.6%) of those who experienced pregnancy coercion reported co-occurring IPV. |
| Falb 2014 | Côte d'Ivoire | - Association between IPV history (independent variables) and RC experience (dependent variable) | - 18.6% reported lifetime partner-perpetrated RC and 49.8% of women reported lifetime partner-perpetrated physical or sexual violence. - Lifetime IPV was associated with a 3.7 increase in odds of reporting RC (95% CI: 2.4-5.8) compared to women who did not report such victimization. |
| Fay 2020 | US | - Association between RC experiences (independent variables) and IPV (dependent variable) | - 47.1% of the women who reported RC screened positive for IPV. - Women reported RC were more likely to report other forms of IPV (35.3% vs 11.9%, P = .02). |
| Grace 2022 | US | - Association between RC experiences (independent variables) and IPV frequency and severity (dependent variables) | - IPV frequency and severity were significantly greater in women who experienced RC on IPV measures (Conflict Tactics Scale (CTS): 6.27 vs 2.67, t = -2.89, p = 0.005 and Women’s Experience of Battering (WEB) Scale: 51.18 vs 33.27, t = -3.50, p = 0.001) |
| Grace 2022 | US | - Co-occurrence of RC and IPV - RC and IPV as distinct but related phenomena | - 16.8% experienced past-year RC, more likely to experience IPV (32.1% vs. 5.7%, p < .001); risk factors included concurrent IPV (AOR = 4.47, 95% CI = [2.06, 9.70], p <.001). - Despite this strong association, the majority of those who experienced RC did not also experience any other form of IPV, lending support to the proposition that RC and IPV are distinct but related phenomena. |
| Hill 2019 | US | - Prevalence of physical and sexual relationship abuse, and nonpartner sexual violence without RC and with RC - Association between RC experiences (independent variables) and physical and sexual relationship abuse, and nonpartner sexual violence (dependent variables) - Association between recent relationship abuse history (independent variable) and RC experience (dependent variable) | - Participants who had RC had higher rates of recent physical relationship abuse (22.7% vs 7.0%, P=.009), recent sexual relationship abuse (27.3% vs 9.1%, P=.019), and recent nonpartner sexual violence (33.3% vs 14.7%, P=.035). - Females who had RC had more than four times the odds of experiencing recent physical relationship abuse (aOR 4.32, 95% CI 2.12–8.79) and recent sexual relationship abuse (aOR 4.24, 95% CI 2.21–8.11); and higher odds of recent nonpartner sexual violence (aOR 2.88, 95% CI 1.33–6.21). - Females who had recent relationship abuse had more than three times the odds of experiencing reproductive coercion (aOR=3.21, 95% CI 1.81–5.71). |
| John 2018 | Ethiopia | - Association between emotional IPV history (independent variable) and RC experience (dependent variable) | - Emotional IPV was strongly predictive of RC (OR: 1.41, CI: 1.14 - 1.74, p<0.05). |
| Katz 2017 | US | - Prevalence of partner physical violence without RC and with RC | - RC and partner physical violence were not independent, χ2(1) = 13.45, p = .01. Among the women who reported past RC, half reported partner violence (50.0%, n = 33). In contrast, just below a quarter of women who did not report past RC reported partner violence (24.8%, n = 39) |
| Kazmerski 2015 | US | - Co-occurrence of RC and IPV - Prevalence of RC without IPV and with IPV | - 16% of respondents reported IPV and 13.5% reported RC in the past 3 months. 4% of all respondents reported both IPV and RC. - 35% of women reporting IPV also reported a history of RC, while 15% of those with no history of IPV reported RC. |
| Kraft 2021 | US | - Co-occurrence of RC and IPV - Association between emotional IPV and RC experience | - 20% of participants had experienced RC; 17.6% reported ever experiencing other forms of IPV, and 57.1% of those who had experienced two or more types of RC had ever experienced other forms of IPV. - A statistically significant bivariate association between RC and other forms of IPV (79.18, p<0.01). |
| McCloskey 2020 | South Africa | - Association between emotional IPV and RC experience - RC as a mediator between IPV and HIV | - IPV is strongly associated with RC (OR: 3.86, 95% CI: 2.89–5.15). - RC acts as a partial or incomplete mediator between IPV and HIV yet exerts independent influence on HIV outcomes. |
| Miller 2010 | US | - Co-occurrence of RC and IPV - IPV as a mediator between RC and unintended pregnancy | - 53% of respondents reported physical or sexual partner violence, 19% reported experiencing pregnancy coercion and 15% reported birth control sabotage. 35% of respondents reporting partner violence also reported RC. - In analyses stratified by partner violence exposure, associations of RC with unintended pregnancy persisted only among women with a history of partner violence. |
| Miller 2014 | US | - Association between RC (independent variable) and unintended pregnancy (dependent variable); IPV not a mediator - Association between IPV (independent variable) and unintended pregnancy (dependent variable); RC not a mediator | - Compared to women exposed to neither RC nor IPV, exposure to recent RC increased the odds of past-year unintended pregnancy, both in the absence of a history of IPV (AOR 1.79, 1.06–2.03) and in combination with a history of IPV (AOR 2.00, 1.15–3.48); history of IPV without recent RC was also associated with unintended pregnancy (AOR 1.80, 1.42–2.26). |
| Muñoz 2023 | US | - Association between RC (independent variable) and physical and sexual IPV (dependent variables) | - Victims of lifetime RC were significantly more likely to experience past-year physical IPV, χ2 (1) = 17.79, p < .001, and past-year sexual IPV, χ2 (1) = 19.80, p < .001, relative to those who did not experience RC. |
| Northridge 2017 | US | - Association between RC (independent variable) and IPV (dependent variable) | - Girls reporting RC were nearly 5 times more likely to report IPV (OR 4.8; 95% CI 2.0-11.8). |
| Paterno 2021 | US | - Association between IPV history (independent variable) and RC experience (dependent variable) | - Past-year RC was associated with past-year IPV (Fisher’s exact test, p = .005). - Odds of past-year RC increased by more than 4 times with past experience of IPV (AOR = 4.74; 95% CI = [1.07, 20.86]). - Women experienced RC identified it as a form of abuse and additionally described experiences of pressure to conceive from the partner’s family. |
| PettyJohn 2021 | US | - Association between RC experience (independent variable) and physical or sexual IPV and multi-perpetrator rape (dependent variables) | - RC was associated with significantly higher odds of physical or sexual IPV (AOR 4.22, 95% CI: 1.60, 11.13), multi-perpetrator rape (AOR 3.56, 95% CI: 1.04, 12.24). |
| Price 2022 | Australia | - Association between DV history (independent variable) and RC experience (dependent variable) | - Women who identified experiencing DV were more likely to identify the presence of RC (21.1%) than women with no other form of DV reported (3.1%), χ2(1, N = 3,117) = 237.86, p < .001. - 34% identified co-occurring RC and only 5.7% of those identified only RC with the absence of other forms of DV. |
| Samankasikorn 2019 | US | - Association between IPV history (independent variable) and RC experience (dependent variable) | - Participants who experienced IPV had a nearly eightfold increased risk of RC (AOR = 7.98, 95% CI [4.68, 13.59]) than their nonabused counterparts. |
| Silverman 2019 | India | - Association between RC (independent variable) and unintended pregnancy and contraceptive use (dependent variables); IPV not a mediator - Association between IPV (independent variable) and unintended pregnancy (dependent variable); RC not a mediator | - Among women experiencing RC, 36% reported that their most recent pregnancy was unintended; these women had 4 to 5 times greater odds of unintended pregnancy and a more than 5 times decreased likelihood of recent use of modern contraceptives than women not experiencing RC, after accounting for effects of demographics and physical and sexual IPV. - Physical IPV also greatly increased the risk of unintended pregnancy. The independent nature of these effects indicates that both RC and IPV relate to reduced reproductive control and that these risks are distinct from one another. |
| Silverman 2020 | Niger | - Association between physical IPV (independent variable) and covert contraceptive use (dependent variable) - Association between RC (independent variable) and covert contraceptive use (dependent variable); physical and sexual IPV as potential mediator | - Exposures of interest (lifetime physical IPV, sexual IPV, and RC) were found to have a prevalence of 8.2%, 5.3%, and 10.2%, respectively. - Unadjusted and adjusted models indicated that physical IPV and RC were associated with covert FP use (vs. no use and vs. overt use). Only physical IPV remained significantly associated with covert use in models including all three forms of violence (AOR: 1.94 vs. any use; AOR: 3.63 vs. overt use). RC was also found to be associated with covert use compared to non-use in the unadjusted model (OR: 2.97, 95% CI: 1.29, 6.80) and after adjusting for covariates (AOR: 3.41, 95% CI: 1.39, 8.34), but did not retain significance in models inclusive of physical and sexual IPV (p = 0.16). |
| Sutherland 2015 | US | - Co-occurrence of RC and IPV | - Of the college women who reported RC (n = 76), 57% also screened positive for IPV (95% CI [2.74, 7.29]). Specifically, 67.9% of the women who reported birth control sabotage and 59.1% of the women reporting pregnancy coercion also reported IPV. |
| Swan 2021 | US | - Association between IPV history and RC experience | - IPV (Sample 1 χ2(1)=10.402, p <0.001; Sample 2 χ2(1)=10.675, p < 0.001) were significantly and positively associated with RC in both samples. |
| Swan 2021 | US | - Association between stalking, sexual harassment, sexual assault, polyvictimization, bullying, psychological partner abuse (independent variables) and RC experience (dependent variable) | - RC was associated with an increased likelihood of experiencing polyvictimization and all interpersonal violence victimization experiences except for physical partner abuse while controlling for demographic factors [stalking (OR = 9.65), sexual harassment (OR = 8.89), sexual assault (OR = 8.72), polyvictimization (OR = 5.40), bullying (OR = 3.49), and psychological partner abuse (OR = 2.31]. |
| Thaller 2016 | US | - Association between RC (independent variable) and forced sex, threats of physical harm, verbal abuse (dependent variables) | - Women who experienced RC were significantly more likely to report forced sex [χ2(1, N = 1,978) = 10.23, p < .001], threats of physical harm [χ2(1, N = 1,970) = 9.06, p < .01], and verbal abuse [χ2(1, N = 1,965) = 10.99, p < .001], though RC was not significantly related to physical violence. |
| Willie 2017 | US | - Association between physical or sexual IPV and RC | - Physical or sexual IPV was directly related to both birth control sabotage (B = 0.40, t = 5.85 p<0.001) and pregnancy coercion (B = 0.33, t = 4.54, p<0.001). |
| Willie 2020 | Mexico | - Co-occurrence of RC and IPV | - Three distinct latent classes: High Physical/High Sexual IPV and High Reproductive Coercion class (16.4%), Low Physical/Low Sexual IPV and Low Reproductive Coercion class (69.8%), and High Physical/Low Sexual IPV, and Low Reproductive Coercion class (13.8%). - Suggest heterogeneity in women's experiences of IPV and RC. IPV can occur without concomitant RC, but our findings suggest that RC tends to co-­occur with high frequencies of both physical and sexual IPV. |
| Wood 2023 | Kenya | - Co-occurrence of RC and IPV | - Within the past three months, over 80% of IPV survivors experienced any RC, and IPV survivors experienced 3.8 RC behaviors on average (sd=3.0), 2.3 pregnancy coercion behaviors (sd=1.8), and 1.5 condom manipulation behaviors (sd=1.5). |

# Table A5. Results on Associations Between Reproductive Coercion and Women’s Health Outcomes.

| First author and year | Country of study | Findings on reproductive coercion association with health outcomes |
| --- | --- | --- |
| Alexander 2021 | US | - Depression: 69% of women with RC reported depression relative to 35% of women without RC (OR = 4.13, 95% CI = [2.19, 7.75]). After adjusting for covariates and primary exposure, RC remained significantly and independently associated with depression (AORRC = 2.86, 95% CI = [1.38, 5.94]). - PTSD: 45.1% of women with RC had PTSD relative to 18.8% of women without RC. After adjusting for covariates and primary exposure, RC remained significantly and independently associated with PTSD (AORRC = 2.41, 95% CI = [1.15, 5.08]). |
| Capasso 2019 | US | - STI: women who experienced PC in the last 3 months were 78% more likely to test positive for an STI than those who did not (aOR = 1.78, 95% CI = 1.10 to 2.90). - Depression: almost twice as likely to have depression (aOR = 1.98, 95% CI = 1.22 to 3.21). Worry about acquiring HIV: more likely to worry about acquiring HIV (aOR = 1.82, 95% CI = 1.09 to 3.04). - Condom use: among women who experienced PC in the last 3 months, odds of not using a condom in the last sexual encounter were 3.45 (95% CI =1.55 to 7.85) those of women not experiencing coercion. - Condom use intentions: women who were coerced had lower intentions of purchasing or using a condom (coefficient, 21.31, P = 0.002). - Condom fear and condom partner barriers: women who were coerced had more fear of negotiating condom use and perceived more barriers to using a condom (coefficients, 3.89 and 5.74, respectively, both P=0.001). |
| Cheng 2021 | Australia | - Contraceptive use: women who disclosed RC were less likely to currently use the oral contraceptive pill (6.3% vs 14.4%; P = 0.007). - After adjusting for demographics and clinic attended, no significant association was found between disclosing RC and the other SRH issues (STI, pregnancy test/options, sexual assault, mental health issues or relationship issues) (P > 0.05). |
| Fay 2020 | US | - Birth weight: have low-birth-weight <2500g neonates at term (17.6% vs 3.1%, P = .03). - Anemia and anxiety: were more likely to report diagnoses of anemia (P = .04) and anxiety (P = .03). |
| Fleury-Steiner 2020 | US | - Perceptions of future violence: women's perceptions of future violence show a trend toward improving prediction of perceptions of future violence (R2 change = .02, p =.08). |
| Grace 2022 | US | - Traumatic brain injury (TBI): women who experienced RC were nearly twice as likely to have also experienced an event that may cause TBI compared to those who had not experienced RC (43.0% vs. 22.9%, p < 0.001). - Depression: RC was significantly associated with increased depression (p = 0.024). - Missing school: more likely to report missing class due to relationship problems (66.3% vs. 44.2%, p = 0.001). |
| Grace 2022 | US | - PTSD: RC was also significantly associated with higher PTSD scores (25.09 vs 18.46, t=-3.47, p=0.001). |
| Grace 2022 | US | - Unplanned pregnancy: women who experienced past-year RC were more likely to have an unplanned pregnancy (34.3% vs. 6.0%, p < .001). - Pregnancy planning: RC remained significantly associated with lower pregnancy planning scores (b= –.154, 95% CI = [–0.301, –0.007], p = .038). The combination of experiencing RC and IPV lowered pregnancy planning scores further (b = –.15, 95% CI = [–0.29, 0.00], p =.052). |
| Hill 2019 | US | - No statistically significant associations between care-seeking behaviors, including pregnancy testing, STI testing or treatment, and any reproductive health visits and RC. |
| Holliday 2017 | US | - Unintended pregnancy: RC was associated with unintended pregnancy in demographic adjusted models (aOR = 1.59, 95% CI = 1.26–2.01) and after controlling for IPV (aOR = 1.47, 95% CI = 1.05–2.05). |
| John 2018 | Ethiopia | - Contraceptive use: a strong negative association between RC and contraceptive use after adjusting for IPV and other factors (OR: 0.77; CI: 0.61-0.97). |
| Jones 2016 | US | - Condom negotiation self-efficacy: adolescents and young adults who reported recent RC had reduced condom negotiation self-efficacy (–0.27 and –0.20, respectively). - Unintended pregnancy: RC was associated with unintended pregnancy among adolescents (odds ratio, 1.1) and young adults (1.1). - STD: RC was associated with reporting an STD among adolescents (1.1) and young adults (1.1). |
| Katz 2017 | US | - Contraceptive use: 81.8% of women who reported RC used at least one reliable form of contraception at last vaginal sex, as compared with 95.5% (n =150) of women who reported no past RC. - Contraceptive and sexual efficacy: women who had experienced RC reported significantly less contraceptive and sexual efficacy (M = 70.94, SD = 10.77) than women who did not report RC (M = 75.70, SD = 9.40), F(1, 221) = 6.67, p = .01. |
| Katz 2020 | US | - Less consistent results regarding associations between partner contraceptive interference (CI) and contraceptive outcomes including efficacy in negotiating condom use and contraceptive use. |
| Kazmerski 2015 | US | - Care-seeking behaviors: recent RC without IPV was associated with increased odds of seeking one (AOR = 2.0, 95 % CI 1.3–2.9) or multiple pregnancy tests (AOR = 2.3, 95 % CI 1.2–4.5), multiple STI tests (AOR = 2.5, 95 % CI 1.5–4.1), or using emergency contraception once (AOR = 2.6, 95 % CI 1.2–5.8) or multiple times (AOR = 2.2, 95 % CI 1.7–2.7). Combined effect of recent IPV and RC increased the odds of seeking multiple pregnancy tests (AOR = 3.6, 95 % CI 3.3–3.8), using emergency contraception multiple times (AOR = 2.4, 95 % CI 1.5–4.1) and seeking STI testing once (AOR = 2.5, 95 % CI 1.6–3.9) or multiple times (AOR = 2.9, 95 % CI 1.02–8.5). |
| Kraft 2021 | US | - Contraceptive use: experiencing two or more forms of RC (vs. not experiencing any RC) was associated with reduced odds of contraceptive use in the past 3 months (aOR = 0.46; 95% CI = 0.24–0.76). - STD: was associated with increased odds of having an STD (aOR= 2.43; 95% CI = 2.35–4.37).  Depression: young women who experienced one or two forms of RC had higher scores on depression than those who did not experience RC (15.11% vs. 14.25% vs. 13.04%, p = 0.01). |
| Liu 2016 | US | - Miscarriage: women were 28 times more likely to have abuse-induced miscarriages if their pregnancies resulted because their abusers did not use birth control (OR = 28.70, p < .05). - Premature births: women were 8 times more likely to report premature births if they were abused because of their use of birth control (OR = 8.340, p < .05). |
| McCauley 2014 | Côte d’Ivoire | - PTSD: women who experienced partner-perpetrated RC were 2.6 times more likely to report past-week probable PTSD (95% CI, 1.7–4.0; P < 0.001), adjusting for exposure to IPV and demographic characteristics. |
| McCloskey 2020 | South Africa | - HIV: RC generates a 42% increase in odds of HIV (adjusted OR 1.42, 95% CI 1.02–1.98). - STI/HSV2: RC is associated with sexually transmitted infection (OR 1.89, 95% CI 1.39–2.55). - Abortion: RC is associated with abortion (OR 2.46, 95% CI 1.41–4.26). - Long‐acting reversible contraception: more women experiencing RC used LARC (OR 1.67, 95% CI 1.04–2.68). |
| Miller 2010 | US | - Unintended pregnancy: RC was associated with unintended pregnancy (AOR 1.60, 95% CI 1.22–2.0). In analyses stratified by partner violence exposure, associations of RC with unintended pregnancy persisted only among women with a history of partner violence. |
| Miller 2014 | US | - Unintended pregnancy: exposure to recent RC increased the odds of past-year unintended pregnancy, both in the absence of a history of IPV (AOR 1.79, 1.06–2.03) and in combination with a history of IPV (AOR 2.00, 1.15–3.48). |
| Northridge 2017 | US | - STD: girls reporting RC were nearly 3 times more likely to have had chlamydia (OR, 2.7; 95% CI, 1.01-7.19). - Communication: girls reporting RC were less likely to have high comfort communicating with their sexual partners (OR, 0.32; 95% CI, 0.1-0.7). |
| Oluseye 2022 | Nigeria | - Consequences of failed abortion include experience of stigma and forced motherhood. |
| PettyJohn 2021 | US | - Unintended pregnancy: RC was associated with significantly higher odds of unintended pregnancy (AOR 5.39, 95% CI: 2.04, 14.25). |
| Price 2022 | Australia | - Mental health: mental health issues disclosed at first contact were more likely to co-occur with RC (36.6%) than in the absence of RC (14.1%), χ2(1, N = 2,458) = 66.83, p < .001. For repeat contacts, disclosure of mental health issues was substantially higher at 60.4% of those experiencing RC compared with 35% disclosing mental health issues and no co-occurring RC. |
| Robinson 2022 | US | - Other health impacts of forced pregnancy may include complications from miscarriage and pregnancy, fetal anomalies that may lead to higher health care burden, and risks to mother's health. |
| Samankasikorn 2019 | US | - In univariate models, RC was significantly associated with increased odds of unintended pregnancy (OR = 2.18, 95% CI [1.38, 3.44]); however, results were nonsignificant after adjusting for sociodemographic factors. |
| Silverman 2019 | India | - Contraceptive use: women who experienced RC were 80% less likely (aOR, 0.2; 95% CI, 0.1–0.4) to have recently used modern contraception. - Unintended pregnancy: had approximately 4 times greater odds (aOR, 3.9; 95% CI, 2.3–6.7) of reporting their most recent pregnancy as unintended. |
| Silverman 2020 | Niger | - RC was found to be associated with covert use compared to non-use in the unadjusted model (OR: 2.97, 95% CI: 1.29, 6.80) and after adjusting for covariates (AOR: 3.41, 95% CI: 1.39, 8.34), but did not retain significance in models inclusive of physical and sexual IPV (p = 0.16). |
| Skracic 2021 | US | - Contraceptive use: women who experienced behavioral types of RC were more likely to currently be using highly versus moderately effective methods (RRR: 22.34, 95% CI: 3.16–157.93, p = 0.002; aRRR: 26.70, 95% CI: 4.59–156.02, p < 0.001) and marginally more likely to be using highly versus low effective contraception (RRR = 5.10, 95% CI: 1.11–23.40, p = 0.04; aRRR = 3.08, 95% CI: 0.97–9.82, p = 0.06). |
| Steinberg 2016 | US | - Mental health: more pre-abortion anxiety and stress symptoms (b = 8.01, SE = 2.20, p < .0005 and b = 6.73, SE = 2.66, p < .05 for anxiety and stress symptoms, respectively). |
| Sutherland 2015 | US | - Contraceptive use: women who experienced RC were more likely to use an injectable birth control method (p = .001). - Abortion: were more likely to report a history of abortion (p<.001). - Unintended pregnancy: were more likely to report a history of unintended pregnancy (p<.001). |
| Swan 2022 | US | - Behavioral health: experiencing RC was associated with increased self-reported anxiety symptoms (M = 10.10 vs. 5.60, t[362] = .08, p < .001), depression symptoms (M = 9.76 vs. 5.88, t[364] = 0.48, p = .002), and alcohol use (M = 4.86 vs. 2.60, t[364] = 0.95, p < .001). |
| Tarzia 2018 | Australia | - PTSD: for women who had experienced coercive behaviour and/or reproductive control without rape or sexual assault, mean PTSD scores was significantly higher (PTSD mean difference = 8.8, 95% CI = 3.9 to 13.7). - Anxiety: mean anxiety scores were significantly higher (anxiety mean difference = 2.7, 95% CI = 1.2 to 4.2). |
| Tomar 2020 | India | - Contraceptive use: women who had experienced RC were less likely to report use of any modern contraceptive (aOR 0.18; 95% CI, 0.09–0.36) in past 1 year. - Contraceptive types: women who faced RC were more likely to use IUDs than pills (aRRR 63.74; 95% CI, 7.42–547.20) and condoms than pills (aRRR 13.9; 95% CI, 2.89–67.30); less likely to use a condom as compared to no contraceptive use (aRRR 0.24; 95% CI, 0.12–0.49); and less likely to use pills than using no method (aRRR 0.02; 95% CI, 0.00–0.07). |
| Willie 2017 | US | - PrEP acceptability: birth control sabotage was directly related to PrEP acceptability (B = 0.19, t = 1.98 p<0.05). |
| Willie 2018 | Liberia | - Prenatal distress: a significant a significant positive association between RC and prenatal distress (B(SE) = 5.127 (1.949), p < .01), and this relationship remained significant after controlling for age, education, relationship status, and employment status. |
| Willie 2019 | US | - Parenting competency: experiencing RC is significantly associated with lower parenting competency (B (SE) = -3.35 (1.22); p= .006). |
| Willie 2021 | US | - STI: a higher proportion of women who reported birth control sabotage had a history of STI diagnosis (76.4% vs. 53.0%; p < .001); (aOR, 2.18; 95% CI, 1.31–3.60; p = .003). |
| Wood 2022 | Ethiopia | - Contraceptive use: past-year pregnancy coercion was associated with a 32% decrease in the odds of modern contraceptive use (aOR = 0.68; 95% CI: 0.53–0.89; Table 4). When examined by severity of pregnancy coercion, odds of contraceptive use only significantly decreased for women who experienced the most severe forms of pregnancy coercion (aOR = 0.59; 95% CI = 0.41–0.83). |
| Wood 2022 | Kenya | - Contraceptive use: women who had experienced more types of overall RC were more likely to use contraception covertly (p=.05); a one unit increase in RC score was associated with the increased relative risk of covert use compared with nonuse (relative risk ratio (RRR)=1.10; p=.05), whereas a one-unit increase in RC score was associated with decreased overt use of contraception compared to nonuse (RRR=0.89; p=.05). |

# Table A6. Results on Women’s Resistance Strategies and Reproductive Coercion Interventions

| First author and year | Country of study | Type of intervention | Findings |
| --- | --- | --- | --- |
| Alexander 2021 | US | - Healthcare providers: screening; - Education; - Other: Interventions that build on existing social support networks to advance safety for Black women and culturally responsive psychoeducational interventions. Adjunctive techniques. | Interventions   - Require specific interventions to address Black women’s psychological well-being using existing social support networks, culturally responsive psychoeducational interventions and adjunctive techniques such as mindfulness and yoga. - Emphasize the need for IPV and RC universal education and assessment. |
| Brenner 2021 | Nicaragua | - Healthcare providers: counseling and provision of contraception; - Social and gender norms | Resistance strategies   - A broad spectrum of coping strategies including acceptance of RC, rationalization of RC, laughter, resistance, sterilization, planning for adoption, threatening the partner, hiding contraceptives, relying on God, buying hormonal emergency contraceptives (HEC), verbally objecting to RC, claiming to be in control and trying to control the partner. - Women rarely succeeded in keeping control over their reproduction over a longer time span.   Interventions   - Ensure access to long-acting contraceptives and implement interventions that challenge traditional masculinities focused on controlling women. |
| Bryson 2021 | US | - Healthcare providers: counseling and provision of contraception; - Policy and legislation; - Social and gender norms; - Education | Interventions   - Provider interventions focus on assessing the ability of all adolescents and young adults to access desired contraceptive method, including LARC, without coercion, and prioritize patient-centered contraceptive counseling focused on individual reproductive goals. - Clinic-level interventions: expanding clinic hours and offering LARC services at convenient locations; same-day LARC placement is recommended; having non-LARC methods in stock. Removing administrative and financial barriers, such as providing confidential care, expeditious provision, and unbiased removal. - Systems-level: ensuring insurance coverage for LARC provision and coverage at novel sites, reviewing confidentiality policies. |
| Buchanan 2021 | Australia | - Healthcare providers: screening; - Healthcare providers: counseling and provision of contraception; - Healthcare providers: referral; - Education | Interventions   - Health providers can provide support by identifying coercive behaviors, fostering empowerment, and collaborating with domestic violence specialists familiar with community resources. - Facilitate additional training for healthcare providers and social workers to jointly recognize and address coercive control. - Health providers can extend empathy, support, and information to women during vulnerable transitions to motherhood, which may be hindered by partners' coercive control. |
| Burton 2015 | US | - Healthcare providers: screening | Interventions:   - Project Connect, a joint venture between the Virginia Department of Health (VDH) Office of Family Health Services and Futures Without Violence, provides training to family planning and home visiting providers on a brief screening procedure and intervention or follow-up preplanning. - Both the training and intervention incorporate principles of trauma informed care, such as recognizing the impact of violence on individual development and coping processes, supporting empowerment through partnership between provider and patient or client, maximizing the individual’s opportunity to choose and navigate a personal path to survivorship, and supporting the individual’s need for safety and non judgmental support in care provision. - Project Connect adapts training for culturally competent care, crucial for addressing trauma in diverse populations. - Evaluation results indicated that providers found training useful, and supervisors appreciated the universal tools and skill set given to participants. - Areas for improvement included increased emphasis on initiating screening, enhancing training for different types of providers, and developing follow-up training. |
| Burton 2021 | US | - Healthcare providers: screening; - Education | Interventions   - Motivations for providers to complete Project Connect screenings in practice include aspects of the patient, the provider, and the workplace. - Patient factors: providers were prompted to screen for IPV and RC when recognizing a patient's youth or risk to children, suggesting a focus on vulnerable groups. - Provider factors: providers' intrinsic professional values, such as compassion and a commitment to patient needs, were key motivators for screening practices. Enhancing protocol uptake may benefit from focusing on providers' perception and integration of risk concerning IPV and RC, beyond mere educational awareness. - Workplace factors: developing a screening culture requires engaging providers at all levels, from protocol development to evaluation, to ensure alignment with professional independence and workload management; normalizing discussions around IPV and RC in clinical practice can help integrate these screenings into routine patient care. |
| Carter 2021 | Australia | - Healthcare providers: screening; - Healthcare providers: counseling and provision of contraception | Interventions:   - Create a confidential and safe environment for patients to disclose reproductive coercion, with clinicians providing non-judgmental, empathetic care, and ensuring access to printed resources and referrals. - Use existing screening tools to sensitively inquire about reproductive health and coercion, tailoring the approach to the patient's situation and ensuring privacy. - Respond to RC by recognizing the patient's expertise, offering person-centered, trauma-informed care, and discussing violence and abuse while respecting the patient's healthcare needs and preferences. - Offer concealment strategies, information on female-led and hidden forms of contraception, STI testing, referrals for sexual assault support services, safety planning, and educational resources. - Understand that contraceptive and pregnancy decision-making can be complex, often influenced by practical or material factors, or a desire to maintain the relationship, even in coercive situations. - Raise awareness may be the primary intervention in the absence of physical or sexual violence. - A multifaceted approach to address women's medical, psychosocial, cultural, and economic needs in strategy development should be explored. |
| Cheng 2021 | Australia | - Healthcare providers: screening | Interventions:   - Opportunity to include RC screening questions in DV screen­ing tools due to the co-occurring nature of DV and RC. - SRH clinicians in particular are well placed to conduct RC screening although they must have adequate training and access to resources to implement screening and respond to women who disclose RC. |
| Cusack 2014 | US / EU | - Healthcare providers: counseling and provision of contraception; - Policy and legislation; - Education; - Vulnerable groups | Interventions:   - Sex educators and healthcare providers should continue to promote condom-use while bolstering non-users’ self-efficacy to safely practice withdrawal method, seek informed consent from partners, and report victimization. - Systemic changes ought to include increased criminalization of nonconsensual insemination, inclusion of nonconsensual insemination in domestic violence legislation, and increased sensitivity and training among police and health-care providers. - Police and legislators should especially focus on accepting victims’ credibility and identifying males and homosexuals as victims of nonconsensual insemination, sexual assault, and domestic violence. - Healthcare providers should increase the attention given to youth, immigrants, and other vulnerable populations. |
| de Bocanegra 2010 | US | - Healthcare providers: screening; - Healthcare providers: counseling and provision of contraception | Resistance strategies:   - End the relationship, covert contraceptive use.   Interventions:   - Exercise caution with joint reproductive decision-making and couple-oriented counseling and assess the male partner's influence on contraceptive and pregnancy choices, and the woman's decision-making independence and comfort with partner involvement. - Train providers to support client self-care in abusive relationships and to address the repercussions of violence post-separation. Incorporate reproductive health issues systematically in the counseling of women at domestic violence shelters. |
| Decker 2017 | US | - Healthcare providers: screening; - Healthcare providers: counseling and provision of contraception - Healthcare providers: referral | Interventions:   - Implement a brief (3–5 minutes) ARCHES intervention through Project Connect to enhanced, universal assessment that supports abuse recognition, provide harm reduction counseling, and facilitate real-time referrals to violence support providers. - Deliver interventions through a provider-facilitated discussion on IPV/RC and distribute palm-sized safety cards that offer health impact quizzes, harm reduction tips like female-controlled contraception, and national resources for violence-related help-seeking. Health centers partner with local IPV service agencies to bolster provider confidence in referring patients. - Results show that trauma-informed discussion of healthy and unhealthy relationships is welcomed by patients affected by IPV and their unaffected counterparts. Those who receive violence-related discussion and/or safety resources felt more confident in their providers’ concern for their safety and ability to respond appropriately to violence. |
| Douglas 2021 | Australia | - Policy and legislation | Interventions:   - Domestic violence support workers and lawyers often view RC as encompassed within existing legislative definitions of DV due to references to coercive or controlling behavior. - However, there's debate over whether the term "RC" is too complex for legislation, with some advocating for the inclusion of specific behaviors to clarify its identification within the DV framework. - Trust development is crucial for disclosures of RC, which face similar challenges as sexual assault claims in the legal realm, often being dismissed as "historical". Delayed reporting and inconsistency can undermine the credibility of survivors, with historically traumatized women faring poorly in legal credibility assessments. |
| Fay 2022 | US | - Healthcare providers: counseling and provision of contraception | Interventions:   - Clinicians should refrain from presuming a lower risk of RC based solely on a patient's use of LARC, as contraceptive choices can vary among those experiencing RC, and understand that diverse contraceptive choices may represent adaptive strategies to counteract a partner’s exercise of power and controls, such as choosing LARC due to a partner's refusal to use condoms. - Acknowledge the important overlap between those most vulnerable to violence and those at high risk for unintended pregnancies, underscoring the need for high-quality, patient-centered contraceptive counseling and support. |
| Galrao 2022 | Australia | - Healthcare providers: screening; - Healthcare providers: counseling and provision of contraception; - Policy and legislation | Interventions   - Screening program at Sexual Health Quarters (SHQ) was well-received by both patients and clinical staff. Of all the women who screened positive for IPV/RC, 61% had been patients at SHQ prior to the introduction of screening and none had their experience of IPV identified in previous consultations. - Implementation took nine months, requiring comprehensive preparation to address barriers and staff concerns, resulting in a supportive and effective screening process. - Advocacy for integrating IPV/RC screening into various healthcare settings where a significant proportion of the patients present with possible indicators for IPV is emphasized to provide quality first contact and avoid repeated survivor narratives. - A call for national and state leadership to train healthcare professionals in IPV/RC identification and respond, and increase emergency counseling resources to ensure accessible and immediate survivor support. |
| Glass 2022 | US | - Healthcare providers: screening; - Healthcare providers: counseling | Interventions:   - myPlan is a free, secure, anonymous mobile and web app designed to assist college-aged women, including racial/ethnic and sexual minority groups, in safety decision-making and planning, available 24/7. - The technology-based intervention has shown efficacy in reducing RC and the risk of suicide in women recently subjected to IPV from a current or former partner. - myPlan is a part of the comprehensive campus and community response to IPV and is intended to complement, not replace, skilled advocacy and counseling services on college campuses and in community settings. |
| Grace 2020 | US | - Policy and legislation; - Vulnerable groups | Resistance strategies:   - Use less detectable contraception, community services, deception and stalling, seek help from family members.   Interventions:   - Clarify legal risks to women seeking health interventions, especially those concerned about undocumented status; and integrate trauma-informed social support into health services, providing universal assistance to women who may be reluctant to request help. - Acknowledge and leverage the inherent strength and resilience of Latina and immigrant women by actively listening and tailoring support to their expressed needs and priorities. |
| Grace 2022 | US | - | Resistance strategies:   - End the relationship, use less detectable contraception, change or hide a method of contraception, had an abortion or did not tell partner about abortion. |
| Hill 2021 | US | - Healthcare providers: screening; - Healthcare providers: counseling and provision of contraception; - Education | Interventions:   - Trauma-Informed Personalized Scripts (TIPS) is an intervention founded on the principles of ARCHES that enhances patient-provider communication about IPV, RC, and contraception needs. It utilizes automated scripts generated from patient responses to a tablet-based survey taken in the clinic's waiting area. While TIPS provides universal education and resources to all patients, it does not depend solely on patient disclosure of IPV/RC, offering a broader approach than traditional screening methods. TIPS aims to facilitate more effective conversations by providing clinicians with tailored scripts that address common barriers such as time constraints and discomfort with sensitive topics. - TIPS, like ARCHES, uses a more comprehensive approach through universal education, providing information and resources to all patients regardless of IPIV/RC disclosure during family planning clinic visits; and provides an alternative to traditional screening approaches that rely heavily on patient disclosure of IPV. - Compared to ‘CUES’’ (confidentiality, universal education and empowerment, and support), an evidence-based strategy to facilitate patient–provider conversations about healthy relationships, TIPS provides an added benefit to improve implementation through tailored scripts with exact phrasing, designed to overcome provider barriers of insufficient time and discomfort. - Results indicate no added benefit of TIPS-Plus (provider scripts + patient messages) compared with TIPS-Basic (provider scripts only), suggesting potential utility of TIPS-Basic in addressing RC and contraceptive uptake. |
| Huslage 2023 | US | - Healthcare providers: screening; - Policy and legislation; - Social and gender norms; - Economic empowerment and financial support; - Education | Interventions:   - Educate healthcare providers on the likelihood and sources of contraceptive pressure, emphasizing patient-centric care and reproductive autonomy. - Include IPV and contraceptive pressure screening in routine care, with a focus on preventing coercion and offering covert birth control solutions, adopting a trauma-informed care approach. - Recognize that RC measures may not capture coercion from other sources, including the healthcare provider, highlighting the need for reproductive justice education and the role of community health workers. - Advocate for policy changes that frame reproductive autonomy and IPV as human rights issues and push for increased state funding for comprehensive social support services, particularly in rural areas. - Promote public support for additional funding by contextualizing IPV as a societal-level issue, despite potential rural resistance to government involvement in IPV matters. |
| Levy-Peck 2016 | US | - Healthcare providers: counseling; - Education; - Other: Advocacy and perpetrator focused interventions | Interventions:   - Encourage professionals, parents, and peers to initiate discussions about the implications of coercive behavior and challenge misconceptions around control and manhood. - Educators and family members should seize opportunities to promote a respectful approach to reproductive choices. - Support survivors who choose to stay in abusive relationships by offering strategies that enhance their safety and future autonomy, such as access to emergency contraception, less detectable birth control methods, and abortion services. - Lobby for removing legislative barriers to achieve women' reproductive autonomy while acknowledging the limitations of the legal system in capturing and addressing RC. Even when coercive acts meet criminal thresholds, they are infrequently reported or lead to legal action. - Include RC in batterer intervention programs. |
| Marie Stopes Australia (Marie Stopes International) 2020 | Australia | - Policy and legislation | Interventions:   - Embed RC in existing and new policies and plans responding to FV, IPV and SV. RC is embedded in the Australian Women’s Health Strategy (2020-2030). Forced sterilisation is mentioned in the Fourth Action Plan of the first National Plan to Reduce Violence Against Women and their Children. - Develop a national Sexual and Reproductive Health and Rights Strategy that addresses interpersonal and structural drivers of RC. - Develop a national healthcare professional training program to address RC in varied healthcare settings. Some online training options offer national access but are tailored to specific jurisdictions or aspects of RC. |
| McGirr 2020 | US | - Healthcare providers: screening; - Healthcare providers: counseling | Interventions:   - Despite low discomfort in discussing reproductive coercion (RC) and few perceived barriers, advocates infrequently engage in RC-related practices. Advocates often address RC reactively, awaiting survivors to initiate discussion. This suggests a need for enhanced training and organizational backing to improve frequency and quality of support provided to survivors. |
| Miller 2010 | US | - Healthcare providers: screening; - Healthcare providers: counseling and provision of contraception; - Healthcare providers: referral | Interventions:   - Healthcare providers recognize male partner RC as a possible cause of contraceptive nonadherence, traditionally attributed to individual factors like lack of knowledge or ambivalence. - Implement a two-step assessment process, starting with a woman’s pregnancy intentions and followed by screenings for RC and IPV. Be attentive to clinical indicators such as inconsistent contraceptive use or frequent requests for emergency contraception. - Use the clinical setting as a platform to educate women about RC and its impact on health and pregnancy risk. Offer information on harm reduction methods like IUDs and injectable contraceptives. - Incorporate safety assessments into options counseling for pregnant women, probing into potential reactions from their partners to their pregnancy status to gauge the risk of violence escalation. - Ensure familiarity with local violence victimization resources and establish a support network within the clinical setting to support healthcare providers in addressing disclosures of violence or coercion. |
| Miller 2011 | US | - Healthcare providers: screening; - Healthcare providers: counseling and provision of contraception; - Healthcare providers: referral | Interventions:   - The intervention focuses on educating clients about reproductive coercion and IPV. - Assessments for IPV and RC are conducted by trained reproductive health specialists and integrated into clinic visits, with discussions tailored to the client's visit purpose, such as partner influence on birth control choices. If IPV or RC is identified, specialists help clients with harm reduction techniques, like hidden birth control methods, and educate clients on local IPV and sexual assault resources and aid in accessing these services, with business-card-sized intervention cards as prompts and resources. - Women in intervention clinics showed a 71% reduction in the odds of pregnancy coercion compared to those receiving standard care, which involves a less comprehensive screening process, and were more likely to report ending a relationship because the relationship was unhealthy or because they felt unsafe regardless of IPV status; suggesting that this intervention may reduce the risk for RC from abusive male partners among family planning clients and support such women to leave unsafe relationships. |
| Miller 2016 | US | - Healthcare providers: screening; - Healthcare providers: counseling and provision of contraception; - Healthcare providers: referral | Interventions:   - ARCHES is a brief family planning provider-delivered intervention combining universal IPV/RC education, harm reduction counseling, and supported referrals to victim services for all clients. - No significant impact on reducing RC or partner violence was found. However, there was an increase in participants' knowledge of partner violence resources and their self-efficacy to apply harm reduction strategies. - Although immediate reductions in partner violence were not observed, participants with higher initial RC scores showed decreased coercion after one year, and usage of the domestic violence hotline increased. |
| Miller 2017 | US | - Healthcare providers: screening; - Healthcare providers: counseling and provision of contraception; - Healthcare providers: referral | Interventions:   - Both providers and patients accepted the integration of IPV and RC education into reproductive health visits positively. Training that leverages patient enthusiasm for disseminating information could alleviate provider apprehension. - ARCHES boosted providers’ confidence in addressing IPV and RC. - Patients reported that the intervention provided vital information, a sense of support, a reduction in feelings of isolation, and empowerment to assist others. - Challenges to implementation included time constraints and the absence of routine prompts for intervention delivery. Incorporating system-level changes like electronic health record prompts and connecting IPV assessment to quality metrics may enhance uptake. |
| Rowlands 2019 | Multi-country | - Healthcare providers: screening; - Healthcare providers: counseling and provision of contraception; - Policy and legislation | Interventions:   - Recognize RC as a possible reason for contraceptive non-compliance in healthcare settings and monitor for behaviors like repeated non-use of contraceptives, multiple requests for emergency contraception, frequent pregnancy or STI testing, and recurrent abortions. - Employ screening tools and interventions to address and mitigate RC. - Ensure private consultations for women, potentially involving safeguarding agencies for vulnerable groups. - Policymakers should incorporate RC awareness into public health strategies to prevent unintended pregnancies, and educational programs should promote understanding of healthy relationships and respectful partner communication. |
| Sheeran 2023 | Australia | - Vulnerable groups | Interventions:   - The lack of interpreter access and the misuse of interpreters, sometimes using perpetrators themselves, has been identified as a significant issue. This not only hinders proper communication but also inadvertently facilitates RC. Ensuring the availability and use of qualified interpreters, specifically trained in RC and abuse, is vital. - Health professionals, police, and interpreters require specific training in understanding and addressing RC. This includes being able to differentiate it from sexual violence and domestic violence to facilitate accurate discussions, disclosures, and appropriate referrals. - There's a need for systematic integration of accessible interpreter services in health and policing, with an emphasis on the routine use of appropriate interpreters and the establishment of relevant policies and procedures. - Further education and clarity are required within the domestic and family violence sectors to effectively identify and respond to RC, separate from other forms of violence. This is necessary to improve service delivery and support for migrant and refugee women. |
| Silverman 2014 | US | - Healthcare providers: counseling and provision of contraception; - Social and gender norms; - Education | Interventions:   - Equip healthcare providers with the skills to offer confidential guidance and support women in defining their reproductive intentions and overcoming obstacles posed by partners. - Ensure a broad spectrum of contraceptives, especially those under women’s control, are widely accessible and affordable to empower women in family planning. - Commit to long-term, multi-sectoral initiatives to dismantle societal norms that endorse male control over female reproductive rights, fostering a culture where coercive behaviors are unacceptable, and develop and scale interventions that transform harmful gender-based attitudes and promote respect for women's reproductive choices across various sectors. |
| Srinivasan 2020 | Australia | - Healthcare providers: counseling | Interventions:   - Women expect healthcare providers to play an important role in responding to reproductive abuse, focusing on taking the problem seriously, reinforcing that the behaviour is wrong, asking about other forms of violence and addressing women’s needs and priorities for contraception and reproductive autonomy. |
| Tarzia 2019 | Australia | - Healthcare providers: screening; - Healthcare providers: counseling; - Policy and legislation | Interventions:   - Trust professional intuition to detect signs of reproductive abuse, which can emerge at any stage of patient interaction, be it during initial contact or in-person consultations. - Establish a secure and supportive environment for women, ensuring privacy during consultations to encourage open discussions about their situations. - Adopt a whole-organization approach that involves efficient time management, reflective practice, and multidisciplinary teamwork to address the medical and psychosocial facets of reproductive abuse. - A pressing need for clear, evidence-based guidelines and training to be developed and implemented to address reproductive abuse in Australian health settings, covering effective identification, response to disclosures, and referral pathways to further support. - Clarifying the roles and collaborative responsibilities of health practitioners to manage reproductive abuse comprehensively. |
| Tarzia 2019 | Australia | - Education; - Social and gender norms | Interventions:   - A need for greater clarity around RC among healthcare providers and how it sits within a broader framework of violence against women, to facilitate cross-disciplinary collaborative responses. - Highlight societal factors contributing to RC, such as community silence and cultural values around motherhood, which may normalize certain aspects of RC. - Propose that RC lies at the intersection of family violence, intimate partner violence, and intimate partner sexual violence, influenced by perpetrator intent and a range of contributing or protective factors. |
| Uysal 2022 | Mexico | - Healthcare providers: screening; - Healthcare providers: counseling and provision of contraception; - Education; - Social and gender norms | Resistance strategies:   - None of the adolescent girls reported using or knowing any strategies to cope with experiences of RC.   Interventions   - Implement health interventions to enhance the capacity, entitlement, and self-efficacy of young girls to manage RC and maintain decision-making autonomy. - Address early sexual initiation, violence, and unintended pregnancy with upstream, community-based interventions to prevent these issues and encourage adolescent girls to seek appropriate healthcare services. - Develop educational models promoting rights-based approaches to safe relationships in Mexican schools, creating safe spaces for girls to disclose violence and access professional support. - Engage boys and men in evidence-based community and school programs to change social norms around RC acceptability. - Utilize interventions like Addressing Reproductive Coercion in Health Settings (ARCHES), proven to increase female-controlled contraceptive use and reduce RC and IPV, indicating potential for adaptation in family planning clinics to meet the specific needs of girls, as evidenced in the U.S. and Nairobi, Kenya. |
| Wellington 2021 | Australia | - Healthcare providers: screening; - Healthcare providers: counseling and provision of contraception; - Healthcare providers: referral; - Policy and legislation | Interventions:   - Healthcare providers indicate the following barriers to responding to RC: lack awareness of RC which hinders early identification and intervention, and feel they have insufficient resources and confidence to effectively respond to RC. Even with training, the lack of referral options creates a barrier to providing comprehensive care. A disconnection between primary care and referral services complicates the support process, especially in rural areas where access to health services is significantly limited. - The need for increased training for clinicians to recognize and address reproductive coercion and abuse. - The establishment of clear, streamlined referral pathways from healthcare to domestic violence/counselling services to improve the support and care for women facing RC. - At a policy level, improve access to abortion, such as telehealth for women living rurally to minimise delay in access, cost of travel and greater privacy and access through the public health system where currently only private is available and unreachable for many women. |
| Zachor 2018 | US | - Healthcare providers: screening; - Healthcare providers: counseling and provision of contraception; - Education | Interventions:   - Two training approaches for healthcare providers on IPV/RC were compared: IPV/RC communication skills workshop involved a simulated patient interaction to build communication skills. It started with a didactic overview covering the prevalence, health consequences, and harm reduction counseling for IPV/RC, along with local and national IPV resources. It then focused on specific communication techniques such as 'Ask-Tell-Ask' and 'N-U-R-S-E' to enhance patient autonomy and provider responsiveness to patients’ emotions. The training included role-playing scenarios with feedback on interviewing strategies. The standard knowledge-based IPV/RC training provided knowledge consisted of the ARCHES intervention. - While the communication skills training improved how providers communicated about IPV and RC, it did not significantly outperform the standard knowledge-based training. In some cases, the standard training was even more effective than the communication skills approach. |
